# Extracellular vesicle miRNA predict FDG‐PET status in patients with classical Hodgkin Lymphoma

**DOI:** 10.1002/jev2.12121

**Published:** 2021-07-15

**Authors:** Esther E. E. Drees, Margaretha G. M. Roemer, Nils J. Groenewegen, Jennifer Perez‐Boza, Monique A. J. van Eijndhoven, Leah I. Prins, Sandra A. W. M. Verkuijlen, Xuan‐Mai Tran, Julia Driessen, G. J. C. Zwezerijnen, Phylicia Stathi, Kevin Mol, Joey J. J. P. Karregat, Aikaterini Kalantidou, Andrea Vallés‐Martí, T. J. Molenaar, Ernesto Aparicio‐Puerta, Erik van Dijk, Bauke Ylstra, Catharina G. M. Groothuis‐Oudshoorn, Michael Hackenberg, Daphne de Jong, Josée M. Zijlstra, D. Michiel Pegtel

**Affiliations:** ^1^ Department of Pathology Cancer Center Amsterdam Amsterdam UMC Vrije Universiteit Amsterdam Amsterdam The Netherlands; ^2^ ExBiome B.V. Amsterdam The Netherlands; ^3^ Department of Hematology Amsterdam UMC Cancer Center Amsterdam, University of Amsterdam Amsterdam The Netherlands; ^4^ Department of Radiology and Nuclear Medicine Cancer Center Amsterdam Amsterdam UMC Vrije Universiteit Amsterdam Amsterdam The Netherlands; ^5^ Department of Genetics Computational Epigenomics and Bioinformatics University of Granada Granada Spain; ^6^ Department of Health Technology and Services Research Technical Medical Centre University of Twente Enschede The Netherlands; ^7^ Department of Hematology Cancer Center Amsterdam Amsterdam UMC Vrije Universiteit Amsterdam Amsterdam The Netherlands

**Keywords:** blood, extracellular vesicles, Hodgkin lymphoma, liquid biopsy, miRNA, response monitoring

## Abstract

Minimally‐invasive tools to assess tumour presence and burden may improve clinical management. FDG‐PET (metabolic) imaging is the current gold standard for interim response assessment in patients with classical Hodgkin Lymphoma (cHL), but this technique cannot be repeated frequently. Here we show that microRNAs (miRNA) associated with tumour‐secreted extracellular vesicles (EVs) in the circulation of cHL patients may improve response assessment. Small RNA sequencing and qRT‐PCR reveal that the relative abundance of cHL‐expressed miRNAs, miR‐127‐3p, miR‐155‐5p, miR‐21‐5p, miR‐24‐3p and let‐7a‐5p is up to hundred‐fold increased in plasma EVs of cHL patients pre‐treatment when compared to complete metabolic responders (CMR). Notably, in partial responders (PR) or treatment‐refractory cases (*n *= 10) the EV‐miRNA levels remain elevated. In comparison, tumour specific copy number variations (CNV) were detected in cell‐free DNA of 8 out of 10 newly diagnosed cHL patients but not in patients with PR. Combining EV‐miR‐127‐3p and/or EV‐let‐7a‐5p levels, with serum TARC (a validated protein cHL biomarker), increases the accuracy for predicting PET‐status (*n *= 129) to an area under the curve of 0.93 (CI: 0.87‐0.99), 93.5% sensitivity, 83.8/85.0% specificity and a negative predictive value of 96%. Thus the level of tumour‐associated miRNAs in plasma EVs is predictive of metabolic tumour activity in cHL patients. Our findings suggest that plasma EV‐miRNA are useful for detection of small residual lesions and may be applied as serial response prediction tool.

## Graphical abstract

Visual abstract was created with BioRender.com.

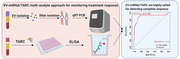

## 1 INTRODUCTION

Classical Hodgkin lymphoma (cHL) is a lymphoid malignancy characterized by an extensive non‐malignant immune cell infiltrate surrounding malignant Hodgkin and Reed‐Sternberg (HRS) cells (Küppers, 2009). Cure rates of cHL following first‐line treatment are high (80%‐90%) (Eichenauer et al., 2017) but intense polychemotherapy regimens often combined with radiotherapy in young cHL patients, can cause severe treatment‐related side‐effects both during treatment, and later in life (Eichenauer et al., 2017; Ng & Van Leeuwen, 2016). The HD18‐trial showed that BEACOPPesc regimes can safely be reduced (de‐escalated) based on a negative interim positron emission tomography scan (iPET), the current gold standard for response evaluation (Borchmann et al., 2017). Incorporating serial measurements of blood‐based tumour biomarkers into real‐time probability models is a promising novel approach for outcome prediction in diffuse large B‐cell lymphoma (Kurtz et al., 2019) that may also improve FDG PET‐guided personalized treatment of cHL patients.

HRS cells secrete cytokines/chemokines to actively recruit immune cells to the tumour microenvironment supporting tumour growth (Steidl et al., 2011; Vardhana & Younes, 2016). HRS cells and stroma secrete immunomodulatory proteins including chemokine CCL17/TARC, CD30, CD163, Galactin‐1 and IGF‐1 which is reflected in patient serum (Cuccaro et al., 2016; Guidetti et al., 2017; Hsi et al., 2019; Jones et al., 2013; Liang et al., 2014; Plattel et al., 2016). In particular, elevated serum TARC (sTARC) levels during and post‐treatment correlate with reduced progression free survival but currently lacks sensitivity and specificity for clinical utility on its own (Guidetti et al., 2017; Hsi et al., 2019; Plattel et al., 2020; Sauer et al., 2013; Viviani et al., 2017). The paucity of HRS cells in cHL challenges biomarker development, however we, and others, have recently shown with shallow whole genome sequencing for CNVs that HRS cell‐free DNA (cfDNA) can be readily detected in the circulation (Amant et al., 2015; Beagan et al., 2021; Spina et al., 2018; Vandenberghe et al., 2015). Finally, 21–23 nucleotide long microRNAs (miRNAs) are released by tumours into circulation and are promising blood‐based biomarkers (Anfossi et al., 2018) of which miR‐21, miR‐494 and miR‐1973 are readily detected in cHL plasma (Jones et al., 2014). In our previous study, we demonstrated that plasma of cHL patients prior to treatment have increased levels of small extracellular vesicles (EVs) with an altered miRNA repertoire (van Eijndhoven et al., 2016). Small EVs and their associated miRNAs can act as mediators of cell‐cell communication promoting tumour progression (Dörsam et al., 2018; Hansen et al., 2016; Zhang et al., 2015) and have strong potential for translation into diagnostic tools for haematological neoplasms (Trino et al., 2020).

Here we performed comparative analyses of EV‐miRNAs, cfDNA (CNV) and sTARC as cHL response biomarkers and extensively evaluated EV‐miRNAs and sTARC in a prospectively collected longitudinal cohort. The levels of EV‐miRNA miR‐127‐3p or let‐7a‐5p in combination with sTARC, has potential to determine therapy‐response during treatment and may be integrated into future dynamic risk assessment models for cHL.

## 2 METHODS

### 2.1 Participants and procedures

Longitudinal plasma EDTA samples (N = 193) derived from 31 patients with cHL were included in this study based on the following inclusion: diagnosed with cHL and older than 18 years and participant of the BioLymph‐trial or Biobank cohort (2018.359). Exclusion criteria were: other malignancies that require treatment simultaneously with the treatment of the lymphoid malignancy. After initial selection, all cHL diagnoses were confirmed according to the criteria of the current WHO classification (Swerdlow et al., 2016) by an experienced haematopathologist. Patient characteristics are found in Table 1, an overview of the number of samples organized by their disease status is listed in Table 2.

**TABLE 1:** Patient cohort at inclusion

	Patient cohort	GLMEM
**cHL patients**	31^*^	31*
de Novo	21	21
R/R	10	10
**Gender**		
Male	23	23
Female	8	8
**Age**, median (range, years)	35 (19‐66)	35 (19‐66)
**Tumour histology** ^**#**^		
Nodular Sclerosis	13	13
Mixed cellularity	4	4
Undefined	6	6
Grey‐zone lymphoma	2	2
**GHSG stage** ^**#**^		
Limited	4	4
Intermediate	4	4
Advanced	17	17
**B‐symptoms** ^**#**^		
Yes	14	14
No	10	10
Unknown	1	1
**Treatment**		
*1^st^ line*		
ABVD+ IFRT	7	7
ABVD/BEACOPPesc	2	2
BEACOPPesc	11	11
BrECADD	1	1
*2^nd^ line*		
BEACOPPesc	1	1
BV‐DHAP + autologous PBSCT	7	7
*3^rd^ line+*		
Pembrolizumab	1	1
BV + allogenic PBSCT	1	1
**EBV + cHL** ^**#**^		
EBV +	9	9
EBV –	14	14
unknown	1	1

^*^25 of the 31 pts had a sample drawn prior to start of treatment.

^#^of the 25 pts with inclusion sample prior to therapy.

R/R = relapsed and/or refractory disease; BV = brentuximab Vedotin; PBSCT = Peripheral blood stem cell transplantation; GLMEM =, generalized linear mixed‐effect model.

**TABLE 2:** Sample cohort

	Sample cohort	GLMEM
**Total amount of samples**	**193^+^**	129^*^
**Pre‐treatment**	**26**	**26**
Active	23	23
PD	2	2
SD	1	1
**During treatment**	**73**	**46**
PR	9	7
CMR	34	28
MR	0	0
PD	1	1
SD	10	10
Response unknown	19	0
**Post‐treatment**	**94**	**57**
PR	2	0
CMR	66	53
MR	2	0
PD	4	1
SD	2	0
Active, relapse	3	3
Response unknown	15	0

+mir24‐3p = 190 and sTARC = 185.

*miR24‐3p = 127 and sTARC = 126.

Complete (metabolic) responders (CMR) samples are defined as blood samples drawn after a negative PET‐CT scan. At the time of draw there are no clinical signs of relapse. MR = mixed response; PD = progressive disease; PR = partial response; SD = stable disease; GLMEM = generalized linear mixed‐effect model.

Small RNA sequencing was performed on 32 samples derived from 14 patients. Matched analyses of cfDNA, EV‐miRNAs and sTARC was performed on 29 longitudinal samples from 17 patients, thereafter it was decided to analyse the total cohort of samples for sTARC and EV‐miRNAs (see Supplemental Figure 9 for Study Design). For the Generalized Linear Mixed‐Effects Model (GLMEM), samples with unknown disease status and potential clinical or technical confounders were excluded (Supplemental Figure 9). A total of 19 blood samples from healthy donors were included in the study as controls (Supplemental Table 10).

### 2.2 FDG‐PET/CT imaging data

FDG‐PET/CT studies were performed using an integrated PET/CT device (Philips Ingenuity TF, Philips Gemini TF 16). FDG‐PET imaging was performed following standard clinical protocol (Boellaard et al., 2015). FDG‐PET scans were performed at staging, after 2 or 3 cycles of induction treatment and at end‐of‐treatment. According to EANM/EARL guidelines, scans were performed 60 min post‐injection (Boellaard et al., 2015). All patients fasted for at least 6 hours prior to FDG injection and blood glucose was confirmed to be below 11 mmol/l. The classification of the blood samples is based on the clinical report of the Nuclear Physician. Blood samples drawn during post‐treatment follow‐up, and after a complete metabolic response (CMR) at end‐of‐treatment FDG‐PET are scored as CMR, unless there are clinical signs of relapse. PET‐negative is classified as a CMR. PET‐positive (i.e. active) encompasses pre‐treatment scans, partial response (PR), progressive disease (PD), mixed response (MR), stable disease (SD), relapsed or refractory (R/R) disease detectable with FDG‐PET positive lesions.

### 2.3 Transfection experiments with Zipper oligo's

Hodgkin lymphoma cell line KMH2 was cultured in RPMI‐1640 (Gibco), supplemented with 10% heat‐inactivated FBS (life science production, S‐001A‐BR, lot: A40319), 100 U/ml penicillin G, 100 μg/ml streptomycin sulfate. LNA miRNA zippers (Qiagen) were designed as described previously published (Meng et al., 2017). Oligo sequences are (5′‐3′): miR‐21‐5p zipper TGA+TAAGCTATTCAACATCAG+TC; miR‐127‐3p zipper CGGATCCGATAGCCA+AGCTC+AGA; miR‐24‐3p AC+TGAGCCAACTGTTCCTGCT+GA; let‐7a zipper CTACTACCTCACAACT+ATACA+AC; and miR‐155‐5p zipper A+TTAGCATTAATAACCCCTAT+CA. As negative control miRCURY LNA miRNA Inhibitor Negative Control A (Qiagen, YI00199006‐ADA) was used. Final concentration for the zippers was 30 nM. The zippers were electroporated into the cells using the Neon™ transfection system (Invitrogen™, MPK5000). Prior to electroporation the cells were counted, centrifuged at 300 g for 5 min, washed with PBS, centrifuged at 300 g for 5 min and resuspended in buffer R (Invitrogen, MPK1025). A total of 1 million cells per reaction were electroporated following Neon's standard protocol with the following settings: pulse voltage 1350 v, pulse width 30 ms, pulse number 1. After the electroporation the cells were plated in 1 ml RPMI‐1640 supplemented with 10% heat‐inactivated FBS. Different cell conditions were counted with trypan blue at 0, 24 and 48 h using a haemocytometer.

### 2.4 Plasma and serum collection

Blood samples were collected in plasma collection tubes (EDTA BD Vacutainer 6 ml) and serum collection tubes (BD Vacutainer SSTII™ Advance 6 ml). Within 1.5 h of collection, isolation of poor platelet plasma was performed. First, the EDTA tube was processed at 900 g for 7 min, then supernatant was spun at 2500 g for 10 min and in some cases an additional spin of 500 g for 10 min was performed. Serum tubes were processed between 30 min and 2 h after collection by centrifuging at 1710 g for 10 min and then supernatant was spun at 500 g for 10 min. Aliquots were stored at ‐80°C until further processing.

### 2.5 Size exclusion chromatography

Size exclusion chromatography (SEC) was used to isolate plasma vesicles in native condition and was performed as described previously (van Eijndhoven et al., 2016). In this study two SEC‐fractions, 9 and 10 (out of 26) were processed and analysed individually to serve as an internal duplicate.

### 2.6 RNA isolation

Total RNA was isolated from SEC fractions using TRIzol® (Thermo Fisher Scientific) according to the manufacturer with some modifications. In brief, 0.75 ml TRIzol® was added to 0.25 ml SEC fractions, incubated at room temperature for 15 min and then stored at ‐80°C for at least 3 h. Prior to isopropyl precipitation 2.5 μg glycogen (Roche) was added. The final RNA pellet was dissolved in 10 μl nuclease free water. For RNA sequencing, RNA from fractions 9–10 from 1.5 ml plasma was pooled in a final volume of 10 μl.

### 2.7 Small RNA sequencing and analysis

Libraries were prepared using Truseq small RNA library prep kit. Small‐RNAseq PE150 was performed on a HiSeq 4000 (Illumina). MiRNA profiling of the sequencing data was performed using sRNAbench (Aparicio‐Puerta et al., 2019; Barturen et al., 2014; Rueda et al., 2015) and miRbase (v22) reference sequences (Kozomara et al., 2019). For differential expression analysis, filtering and normalization of the data was performed using the R package EdgeR (Robinson et al., 2010). Libraries were normalized using the Trimmed mean of M‐values method (TMM) (Robinson & Oshlack, 2010). MicroRNAs with less than 1 LogCPM (Counts per Million) in half of the samples were excluded from the differential expression analysis. Logistical regression was performed using CPM‐data from the five candidate miRNAs.

### 2.8 Multiplex stemloop qRT‐PCR

MiRNA qRT‐PCR was done as described before (van Eijndhoven et al., 2016). In brief; equal volumes of RNA (3 μl) were reverse transcribed with TaqMan® MicroRNA Reverse Transcription kit (Thermo Fisher Scientific). Four RT‐primers (miR127‐3p (ID000452), miR155‐5p (ID002623), miR21‐5p (ID000397) and let7a‐5p (ID000377)) were used in a validated multiplex reaction. miR24‐3p (ID000402), miR10b‐5p (ID002218) and miR150‐5p (ID00473) were performed in separate RT‐reactions. cDNA was subjected to 40 cycles of 95°C for 15 s and 60°C for 1 min on a ABI 7500 Fast system and data was analysed using 7500 Software v2.3, and for comparison of miRNA levels the Cq threshold was set at similar levels in different PCR runs.

### 2.9 Shallow whole genome sequencing (sWGS) of cfDNA

Cell‐free DNA (cfDNA) was isolated on the QiaSymphony (QIAGEN) according to the manufacturers instruction. The concentration of all cfDNA samples was measured by fluorometric quantification using the Qubit Double Stranded DNA High Sensitivity Assay Kit (dsDNA HS) (ThermoFisher Scientific, MA, USA). The cfDNA samples underwent library preparation with the KAPA HTP Library Prep Kit (Roche Sequencing, CA, USA) according to the manufacturer's protocol with 4–6 PCR cycles in 24 out of 29 samples. The other four samples were sequenced without PCR‐amplification. Libraries were measured using TapeStation, followed by generation of an equimolar sequencing pool in a final target concentration of 3 nM. The sequencing pool underwent a paired‐end 150 sequencing protocol on an Illumina HiSeq4000 sequencer. Data analysis was performed as previously described (Beagan et al., 2021). Processing was performed with 100 kbp‐bins and without size selection. All segments > 3 megabases in the tumour profiles were compared with the matching segment values for the healthy normals, and a segment was called aberrant if its z‐score > 5. A tumour sample was called aberrant if at least one segment was called aberrant. Copy number profiles were generated using QDNAs Equation (v. 1.1.12).

### 2.10 TARC analysis

Serum CCL17/Thymus and activation‐regulated chemokine (TARC) was measured using a double antibody sandwich ELISA (Human CCL17/TARC DuoSet ELISA; R&D Systems Europe, Ltd., Abingdon, OX, UK cat#DY364, lot# P129843 and P168719) following standard protocol.

### 2.11 Statistics

A Kolmogorov‐Smirnov test was used to test for Gaussian distribution, followed by parametric or non‐parametric tests. Standard deviation and Coefficient of Variation (CoV) of TARC values were calculated with ELISA duplicates. Samples with CoV > 15% were repeated. To correct for different plasma inputs (1.0 or 1.5 ml), qRT‐PCR data was normalized to the mean of the complete metabolic response (CMR) during and post‐treatment. Relative change is calculated using 2^–∆∆Cq^ in where the ∆∆Cq is the difference between the Cq‐value measured at that timepoint minus the Cq‐value of the CMR group. Generalized Linear Mixed‐Effects model (i.e. GLMEM) using R‐package lme4 (Bates et al., 2015) was used to analyse EV‐miRNAs as a combined biomarker panel and backward selection was used to determine the optimal model. sTARC was log‐transformed and then relative change was calculated by 2^∆sample – mean(CMRgroup)^ prior to running the GLMEM. Youden‐index and Closest‐top‐left were used to determine the sensitivity, specificity, negative predictive value and positive predictive value (Bates et al., 2015). The pROC R package was used to make the ROC‐curves and calculate the area under the curve (Robin et al., 2011). Bootstrap was used to calculate the optimism‐corrected performance (in terms of the area of the curve) (Efron & Tibshirani, 1993; Harrel, 2015; Neeman, 2009; van Houwelingen & Le Cessie, 1990). Spearman correlation matrix was performed using the corrplot R package (Wei & Simko, 2017). The Gene‐miRNA network analysis was performed with miRNet (Fan & Xia, 2018; Fan et al., 2016). A shortest path filter on all network nodes was used to reduce the amount of miRNA‐gene interactions. Analyses were performed using Graphpad Prism 9 software version or R‐Studio v1.1.463.

### 2.12 Data sharing statement

Individual participant data that underlie the results reported in this Article, after de‐identification, will be available together with the study protocol. This will be immediately following publication. Data will be available for researchers who provide a methodologically sound proposal that fits within the scope of the study and is in accordance with the informed consent of the patients. Proposals should be directed to d.pegtel@amsterdamumc.nl ; to gain access, data requestors will need to provide a draft of a data access agreement that will be evaluated. The small RNA sequencing data can be found in the SRA database under accession‐number: PRJNA742213.

### 2.13 Study approval

Samples were collected in the BioLymph‐study (2017‐2019, VUmc METc registration number: 2017.008). The study was registered in the Dutch CCMO‐register (toetsingonline.nl, NL60245.029.17) and is being conducted in accordance to the Declaration of Helsinki (7th revision, October 2013) and in accordance with the Medical Research Involving Human Subjects Act (WMO). A second set of samples (2014‐2017), prior to the BioLymph study, has been collected through biobanking and are registered at the Biobank approval committee of VUmc, Amsterdam (2018.359).

## 3 RESULTS

### 3.1 Small RNAseq of plasma EV fractions reveal miRNAs that distinguish FDG‐PET positive cHL patients from complete responders

**FIGURE 1:**
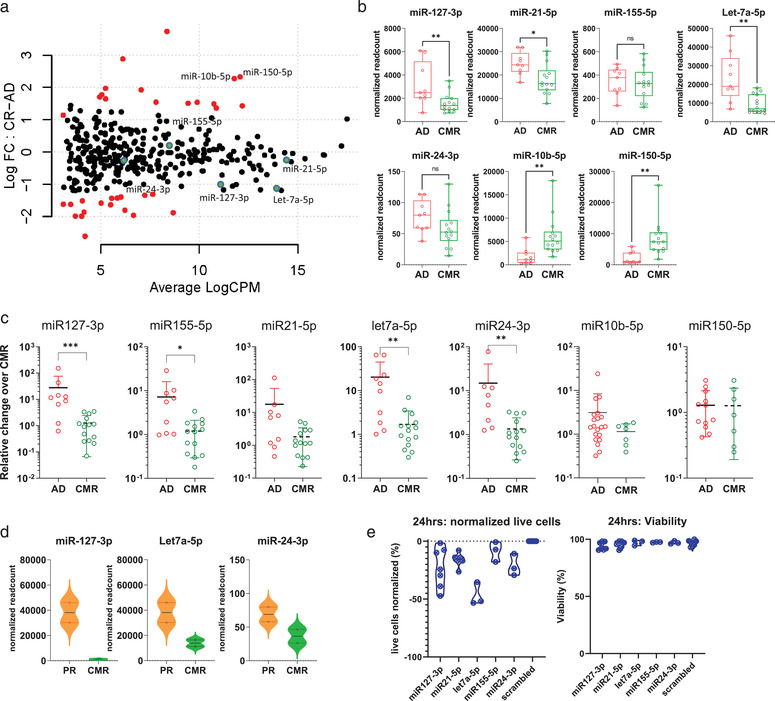
plasma EV miRNAs as determined by small RNAseq and qRT‐PCR distinguish cHL patients with active disease from complete responders. (a) Mapplot of differential expression analysis comparing active disease samples (pre‐treatment plus partial responders) (*n *= 9) to complete responders (*n *= 10). On the y‐axis the normalized log fold change (log FC) is shown and on the x‐axis the average log count per million (CPM, i.e. normalized read counts). Each dot is a miRNA in the analysis. Red dots are significantly differently miRNAs. The green dots highlight the five candidate miRNAs. (b) Normalized read counts per EV‐miRNAs as determined by RNAseq. AD = active disease which is defined as FDG‐PET positive cHL, including partial responders (*n =* 9). CMR = complete metabolic response (*n =* 14). Every point is a single sample. Boxes show the 25%–75% percentile; whiskers show the minimum‐maximum; and lines represent the median. *P < 0.05, ** *P* < 0.01, *** *P *< 0.001, **** *P *< 0.0001 (Mann Whitney test). (c) qRT‐PCR of miR‐127‐3p, miR‐155‐5p, miR‐21‐5p, Let‐7a‐5p, miR‐24‐3p, miR‐10b‐5p and miR‐150‐5p cHL patients samples. For the qRT‐PCR plot of miR‐127‐3p, miR‐155‐5p, miR‐21‐5p, Let‐7a‐5p, miR‐24‐3p, active disease samples (*n* = 9), complete metabolic responders (*n *= 14) are depicted. For miR‐150‐5p: Active disease (*n* = 12), complete metabolic responders (*n *= 8). For miR10‐5p: Active disease (*n* = 20), complete metabolic responders (*n *= 7). *P < 0.05, ** *P* < 0.01, *** *P *< 0.001, **** *P *< 0.0001 (Mann Whitney test). Relative change is the qRT‐PCR normalized to the mean of the CMR‐group. (d) Normalized read counts from the plasma samples at time at interim‐PET. CMR = complete metabolic response and PR = partial response. (e) Cell count and viability data from KMH2 cell line electroporated with miRNA‐zippers, 24 h post electroporation. miRNA target of the zipper depicted on the x‐axis. In the first plot, the absolute live cell counts at 24 h normalized to the live cell count of the negative control are depicted on the Y‐axis in percentages. Every point is the mean of the two technical replicates of one experiment. Viability is the percentage of live cells divided by the total amount of cells present.

We showed previously that HRS cells secrete miRNAs via EVs and that serve as potential cHL disease biomarkers (van Eijndhoven et al., 2016). To confirm these findings, using a validated size‐exclusion chromatography protocol (Boing et al., 2014), we purified 32 plasma EV fractions from 14 cHL patients (pre‐treatment, during and post‐treatment samples) and performed an extensive analysis of the full miRNA content by small RNA sequencing (Aparicio‐Puerta et al., 2019; Barturen et al., 2014; Rueda et al., 2015). Differential expression (DE) of normalized reads counts (CPM) revealed that 33 miRNAs are significantly increased or reduced in active (PET‐positive) disease compared to CMR, although many were lowly abundant casting doubt on their biological significance (Figure 1a, full DE‐list in supplemental data 1). However, such differences were absent when comparing samples from newly diagnosed cHL patients and R/R patients (two differentially expressed miRNAs: supplemental Figure 1, Full list: supplemental data 1), suggesting disease status, and not treatment itself, underlie the observed differences.

In agreement with NGS data from healthy versus active disease (van Eijndhoven et al., 2016), our pre‐selected candidates miR‐127‐3p, miR‐21‐5p, let‐7a‐5p and miR‐24‐3p consistently showed reduced normalized read‐counts in patients with CMR (although miR‐24‐3p does not reach significance). In addition, in this NGS‐based comparison miR155‐5p read‐counts do not differ between active disease and CMR (Figure 1b) as we also observed before and is probably due to technical bias. The DE analysis identified miR‐150‐5p and miR‐10b‐5p, as elevated candidate markers after treatment in responders (Figure 1a). Next, we measured the levels of pre‐selected and novel candidate markers in the same samples with qRT‐PCR. While we could validate miR‐127‐3p, miR‐155‐5p, let‐7a‐5p and miR‐24‐3p by qRT‐PCR, miR‐150‐5p, miR‐10b‐5p levels, did not differ between active disease and CMR groups as determined by qRT‐PCR (Figure 1c). Unsupervised hierarchical clustering groups PR with active cHL and separated PR from CMR samples (Supplemental Figure 2A). Logistical regression further confirmed that the EV‐miRNA‐panel discriminates PET‐positive from CMR samples (supplemental Figure 2B).

Notably, at interim analysis the three significant miRNAs (i.e. miR‐127‐3p, let‐7a‐5p and miR‐24‐3p), had higher normalized read‐counts in patients with PR in comparison to during treatment (at iPET) CMR (Figure 1d). Fittingly, selective impairment of miRNA activity in HRS (KMH2) cells cultured *in vitro* transiently reduced cell growth most strongly when miR‐127‐3p, miR‐24‐3p and Let‐7a‐5p were inhibited (Figure 1e & Supplemental Figure 3). Analysis with miRNet (Fan & Xia, 2018; Fan et al., 2016) suggests that the miRNA panel controls p53, JAK‐STAT and B cell receptor signalling pathways. This data point to a functional role for this miRNA panel in lymphomagenesis that target the lymphoma related genes *MYC, TNFAIP3, SOCS1, BCL6* or *PRDM1* (as indicated in blue; Supplemental Figure 3B).

Overall, we conclude that our pre‐selected EV‐miRNA biomarker panel has potential for response assessment in cHL patients undergoing treatment. Based on this and previous data (van Eijndhoven et al., 2016), we decided to further investigate our initial panel of EV‐miRNAs using a qRT‐PCR‐based detection assays which can be easily adapted in clinical practice.

### 3.2 EV‐miRNAs and sTARC outperform CNV detection in cfDNA for blood‐based cHL detection

**FIGURE 2:**
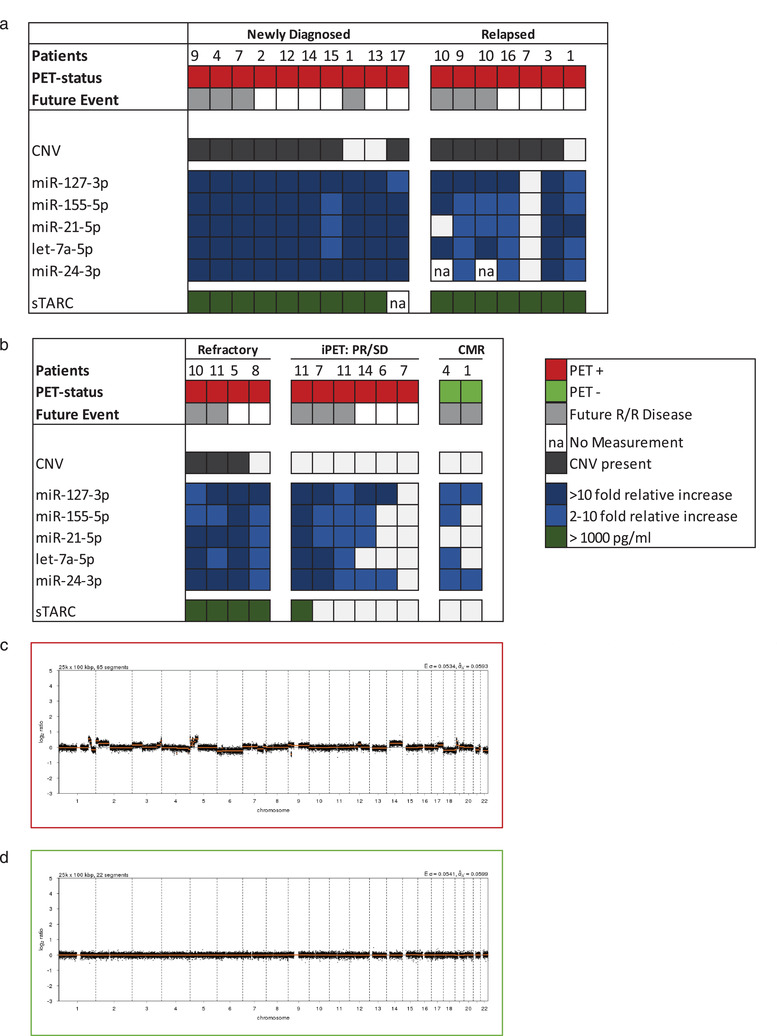
EV‐miRNAs and sTARC outperform CNV detection in cfDNA for blood‐based cHL detection. Overview of the matched analyses of copy‐number variations (CNV), EV‐miRNAs and sTARC in 29 samples from 17 cHL patients in (a) and (b). (a) Depicts the pre‐treatment samples from newly‐diagnosed or relapsed cHL patients and (b) depict the during and post‐treatment samples. Colour legend is depicted in the figure. (c) Copy number profile of cHL patient, analysed using QDNAseq. On the x‐axis the different chromosomes are depicted, and on the y‐axis the normalized log2 ratio is shown. This is a representative example of a profile scored positive. (d) Copy number profile of cHL patient, analysed using QDNAseq. On the x‐axis the different chromosomes are depicted, and on the y‐axis the normalized log2 ratio is shown. This is a representative example of a profile scored negative. In figure (a) and (b) a negative profile is depicted as a white box.

To investigate whether PCR‐based detection of our candidate EV‐miRNAs has similar potential as sTARC and cfDNA CNV analyses, we performed a comparative analysis in 29 samples from 17 cHL patients (Figure 2). Using an unbiased calling algorithm we detected CNVs in 8 out of 10 newly diagnosed cHL cases (Figure 2a). In relapsed (*n *= 7, Figure 2a) and treatment‐refractory (*n *= 4, Figure 2b) cases we detected CNVs in 9 out of 11 cases, while EV‐miRNAs were able to detect an > 2‐fold relative increase (relative to mean of the CMR samples) in 10 out of 11 cases and sTARC detected all 11 cases (> 1000 pg/ml). However, the added value of EV‐miRNAs is most apparent in during treatment samples. EV‐miRNAs remain elevated above CMR levels in 5 out of the 6 cases, including all 3 cases with residual disease at end‐of‐treatment or those that progressed throughout treatment (refractory cases, Figure 2b). In contrast, none of these samples had detectable CNVs and only 1 had elevated TARC. Representative examples of CNV profiles scored as positive and as negative are provided in Figure 2c (positive) and 2D (negative). In addition, EV‐miRNAs and sTARC outperform CNVs at detecting R/R cases. For these reasons we selected EV‐miRNAs and sTARC for longitudinal analyses.

### 3.3 The miRNA panel measured by qRT‐PCR in plasma EVs is consistently elevated in cHL patients with active disease that drop in complete responders

**FIGURE 3:**
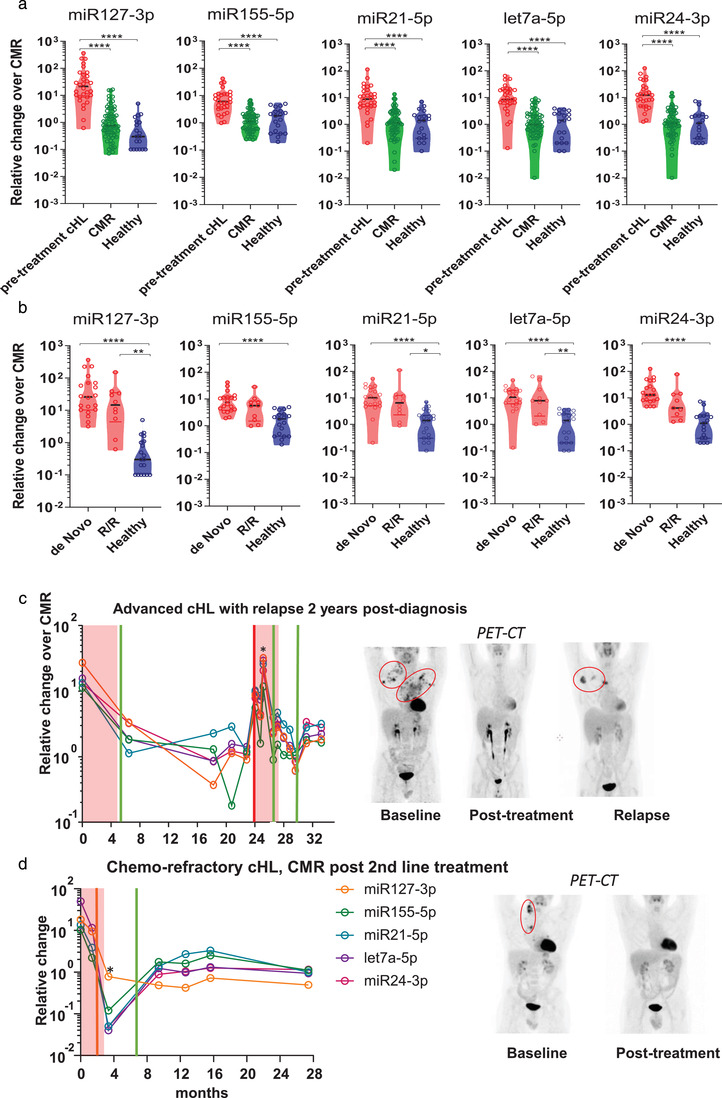
EV‐miRNAs stably differentiate FDG‐PET active cHL patients from complete responders and healthy donors. (a) qRT‐PCR analysis of EV‐associated miR‐127‐3p, miR‐155‐5p, miR‐21‐5p, Let‐7a‐5p, miR‐24‐3p in FDG‐PET active cHL samples prior to treatment (*n* = 30) versus post treatment complete metabolic responders (*n *= 66) and healthy donors (*n *= 19). Each point in violin plot is an sample, horizontal black line is the median. Relative change is the qRT‐PCR normalized to the mean of the CMR‐group. *P < 0.05, ** *P* < 0.01, *** *P *< 0.001, **** *P *< 0.0001 (Kruskall Wallis test). (b) qRT‐PCR of miR‐127‐3p, miR‐155‐5p, miR‐21‐5p, Let‐7a‐5p and miR‐24‐3p in newly diagnosed cHL patients (*n *= 20) versus relapsed and/or refractory (R/R) patients pre‐treatment (*n *= 10) and healthy donors (*n *= 19). Each point in violin plot is an sample, horizontal black line is the median. Relative change is the qRT‐PCR normalized to the mean of the CMR‐group. (c) EV‐miRNA levels (measured by qRT‐PCR) in longitudinally collected samples of a newly diagnosed cHL patient who presented with advanced stage and relapsed 24 months after inclusion and first‐line treatment. The asterisks depict a timepoint where the patient was hospitalized with neutropenic fever and symptoms of respiratory tract infection. (d) EV‐miRNA levels (measured by qRT‐PCR) in longitudinally collected samples of a cHL patient who presented with refractory disease during first‐line treatment as in (c). The sample with an asterisk is taken 14 days post BEAM and autologous stem cell transplantation. Image panels depicts the FDG‐PET data in Maximum intensity Projections (MIPs). The red boxes in the graphs (c) and (d) depict the treatment phase(s). The x‐axis depicts the time in months after inclusion. The vertical green lines in the graphs depict the timepoint where the patient has no disease activity based on PET‐CT. Vertical orange line is a timepoint in where the patient has a partial response on PET‐CT and the red line is the moment in where PET‐CT shows a relapse.

Having demonstrated with small RNAseq and qRT‐PCR that our EV‐miRNA panel is associated with disease activity and cHL treatment response, we measured their absolute levels in 193 samples from 31 cHL patients and 19 non‐serial healthy donor samples by qRT‐PCR. The average level in pre‐treatment cHL samples (*n *= 30) is significantly increased compared to that of healthy donors and post‐treatment samples (*n *= 66) from patients who had a CMR at end‐of‐treatment FDG‐PET (Figure 3a). The qRT‐PCR‐data was normalized to the mean of the CMR‐group which mitigates the risk of losing information when using a personalized method compared pre and post levels within the same patient (as specified in the methods). Since the pre‐treatment EV‐miRNA levels in R/R cHL patients (*n *= 10) did not differ from previously untreated newly diagnosed cHL patients (*n *= 20, Figure 3b), recurrence of disease activity may be detectable with these selected EV‐miRNAs.

Encouraged by these results, we evaluated whether the EV‐miRNA panel is suitable for individualized monitoring of response to therapy in 2 exemplary patients. The first patient (Figure 3c) is a 39‐year old male whom presented with advanced stage cHL and high EV‐miRNA levels. Successful treatment with 6 cycles BEACOPPesc led to a typical drop in EV‐miRNA levels relative to those of CMR that remained in this ‘healthy control group range’ for nearly 2 years. However, after 24 months at the time of a relapse, all EV‐miRNA levels increased. We measured an > 10 fold relative increase in all five EV‐miRNAs (range: 11.73 – 31.93) during a period of neutropenic fever when circulating white blood cells counts are low. Early during the 2^nd^ line treatment EV‐miRNAs level decreased again and stayed low post‐treatment.

The second patient example (Figure 3d) is a 31‐year old male cHL patient who presented with PD after 1^st^ line treatment at inclusion (t = 0). During 2nd line treatment the patient had a PR at interim assessment and CMR at end‐of‐treatment. The 5 EV‐miRNA panel remained low during and post‐treatment. Two weeks post BEAM and autologous stem cell transplantation (i.e. pan‐cytopenic period during stem cell engraftment in the bone‐marrow), the EV‐miRNA levels were very low, which we also noticed in 3 similar additional cases. We conclude that EV‐miRNAs are significantly elevated (*P* < 0.0001) in pre‐treatment cHL patient plasma in comparison to the CMR‐samples. These levels drop in patients with CMR to healthy donor levels. This suggests utility for minimally‐invasive interim treatment response evaluation with EV‐miRNAs.

### 3.4 EV‐miRNAs of complete responders early during treatment are indistinguishable from post‐treatment levels and are not affected by bone marrow depression or organ function

**FIGURE 4:**
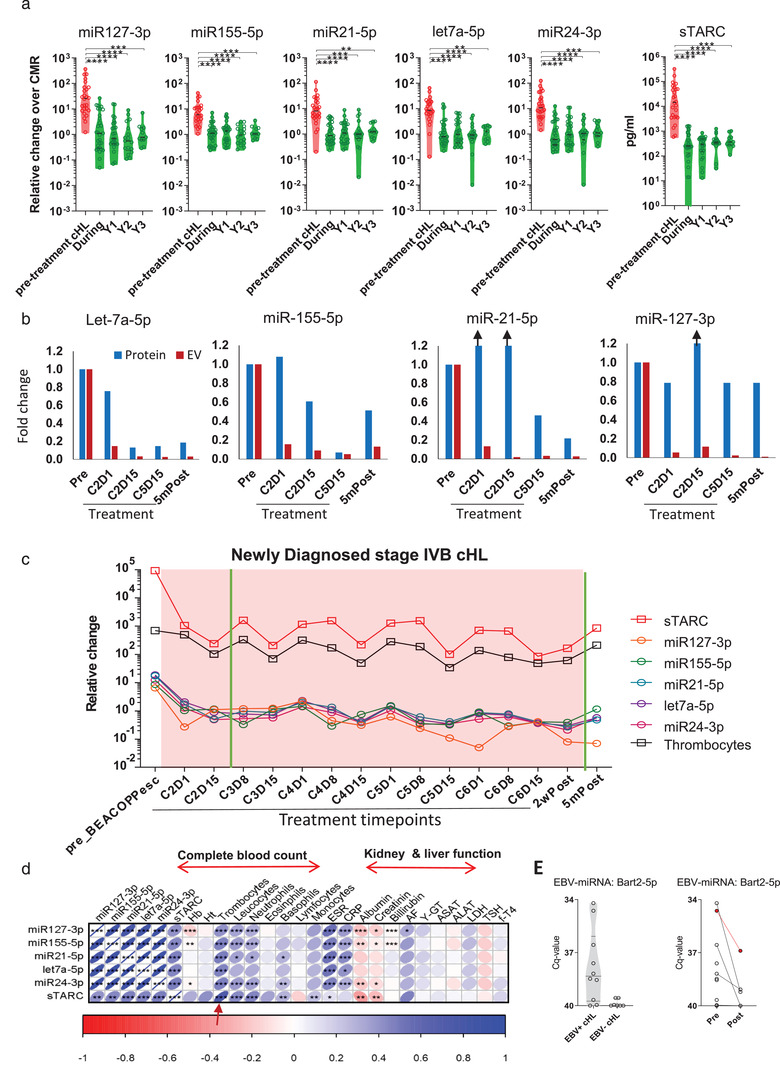
EV‐miRNAs of complete responders decrease early during treatment and are indistinguishable from post‐treatment levels. (a) qRT‐PCR of miR‐127‐3p, miR‐155‐5p, miR‐21‐5p, let‐7a‐5p, miR‐24‐3p and sTARC analysis by ELISA for cHL patient samples pre‐treatment (*n *= 30), during treatment samples from complete responders (during), and year 1 through 3 (Y1‐3) post‐treatment follow up samples. Line represents the mean and upper whisker is the standard deviation. Relative change is the qRT‐PCR normalized to the mean of the CMR‐group. * *P *< 0.05, ** *P *< 0.01, *** *P *< 0.001, **** *P *< 0.0001 (Kruskall‐Wallis test). (b) qRT‐PCR of miR‐127‐3p, miR‐155‐5p, miR‐21‐5p and let‐7a‐5p on the size exclusion chromatography EV‐ and protein‐fractions. Different timepoints from the same patient are analysed. The bars depict the log‐fold change in respect to the pre‐treatment sample. Black arrows depict samples which are higher than 1.2 fold change. Full graph of this patient is shown in Supplementary Figure 4. C = treatment cycle, D = day. (c) Longitudinal EV‐miRNA and sTARC profile of an advanced stage, newly diagnosed cHL patient who receives six cycles of BEACOPPesc. The red boxes depict the phase in where the patient receive treatment. The y‐axis depicts the time in months after inclusion. The x‐axis for sTARC is pg/ml and for thrombocytes 10^9^/litre. The vertical green lines in the graph depict the timepoint in where patient showed no FDG‐activity on PET‐CT. (d) Correlation matrix of qRT‐PCR miRNAs and sTARC data versus laboratory parameters measured at the same day of blood draw. Number of samples per correlation are depicted in supplemental Table 11. Hb = haemoglobin; Ht = haematocrit. (e) qRT‐PCR analysis of EBV‐miRNA Bart2‐5p in plasma (protein‐fraction) of pre‐treatment cHL samples (left). qRT‐PCR analysis of Bart2‐5p pre‐ (*n *= 8) and post‐treatment (*n* = 7) in EBV+ cHL (right). The patient in red is diagnosed with a relapse 1 year after the post‐treatment sample.

Of the 193 EV‐miRNAs panel analysed samples, 135 longitudinal samples were from 25 CMR patients post‐treatment. We observed a rapid and stable decrease in the EV‐miRNA panel during treatment. Notably, the levels in CMR remain low during a 3‐year follow‐up (28 pre‐treatment samples with 82 CMR follow‐up samples; Figure 4a). sTARC again followed a similar pattern to the EV‐miRNA panel (Figure 4a). These data confirm the suitability of our 5‐miRNA panel for response assessment in cHL patients by qRT‐PCR.

In our earlier work, we showed the relevance of detecting EV‐miRNAs over cell‐free miRNA in total plasma when comparing cHL patients pre‐treatment with healthy individuals (van Eijndhoven et al., 2016). To show the importance of measuring in the EV‐fractions over protein‐associated miRNAs in pre‐treatment versus post‐treatment CMR cHL patients, we measured four candidate miRNAs longitudinally in two patients at multiple timepoints (Figure 4b and Supplemental Figure 4). The miRNA levels decrease substantially in EV‐fractions upon treatment and remain low in this CMR patient. However, when measuring the same miRNAs in the protein‐fractions of the same patients, the difference between pre and post‐treatment is less obvious.

To investigate the possibility that the EV‐miRNA and sTARC signals are partly or completely derived from non‐tumour sources, we calculated correlations with circulating blood cells, thrombocytes and assessed a potential link with liver and kidney function. In Figure 4d, we present an example from a newly diagnosed, advanced stage cHL patient, who received 6 cycles of BEACOPPesc. This is the same patient as in Figure 4b. Interestingly, during treatment cycles fluctuating sTARC levels correlate strongly with thrombocyte levels (Figure 4c, black line). Using the complete cohort data, we observed weak but significant relationships between miR‐127‐3p, miR‐155‐5p, miR‐24‐3p and sTARC with albumin and creatinine (*N* = 193, Figure 4d). Importantly, this correlation was not apparent in CMR samples (*n *= 100, Supplemental Figure 5), suggestive of a treatment‐related correlation. In contrast, sTARC correlated with leucocytes (r = 0.44), neutrophils (r = 0.45), basophils (r = 0.29), monocytes (r = 0.24), and as suspected with thrombocytes (r = 0.73) suggesting that sTARC levels are susceptible to treatment‐related changes of the bone marrow (Figure 4d). Moreover, both the EV‐miRNA panel and sTARC did not correlate with age of the patient (Supplemental Figure 6). sTARC levels is significant different between males and females in complete responders (*P* = 0.0001, Supplemental Figure 6), as is the case with miR‐155‐5p and let‐7a‐5p but not with the other three EV‐miRNAs.

Interestingly, we could detect EBV‐encoded BART2‐5p in plasma‐protein fractions in EBV‐positive cHL cases before treatment (except in one case that relapsed, Figure 4e). This observation confirms our suspicion that miRNAs expressed by HRS cells can enter circulation but that their levels are low. Despite the uncertainty regarding their exact tissue and cellular origin, we conclude that the fluctuating levels of our EV‐miRNA panel in plasma EVs from cHL patients undergoing treatment relates to the presence of disease lesions with HRS cells rather than fluctuations in blood cell counts and organ function induced by the treatment itself.

### 3.5 EV‐miRNAs correctly classify FDG‐PET status in cHL patients and has potential for individual therapy response monitoring

**FIGURE 5:**
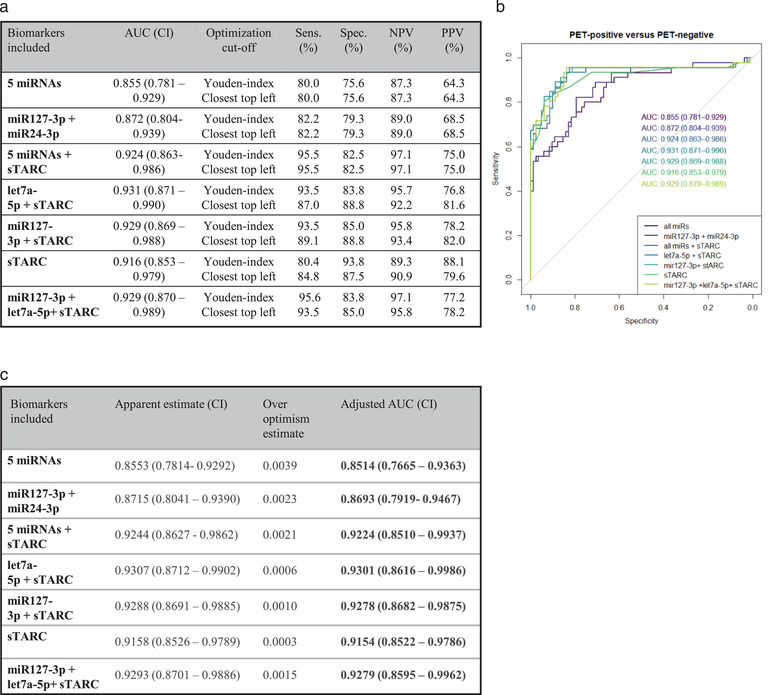
EV‐miRNAs correctly classify FDG‐PET status in cHL patients and has potential for individual therapy response monitoring. (a) Table with the Area Under de Curve (AUC), sensitivity (Sens.), specificity (Spec.), negative predictive value (NPV) and positive predictive value (PPV) of the Generalized linear mixed effect models (GLMEM) as depicted in (b). Cut‐off to determine sensitivity and specificity is done using a Youden‐index or closest‐top‐left calculation. (B) ROC curve generated by GLMEM on 129 cHL samples pre‐, during and post‐treatment. (c) Bootstrap validation of GLMEM models, over optimism estimate and adjusted AUC are depicted in this table. CI = confidence interval.

To evaluate the diagnostic potential of our EV‐miRNA panel and sTARC in longitudinal samples of a cHL patient cohort (*n *= 129) reflecting real‐world academic hospital clinical care, we performed a generalized linear effects model (GLMEM). The inclusion and exclusion criteria for the model are described in Supplemental Figure 9. The complete five EV‐miRNA panel yielded an AUC of 0.855 (CI 0.781‐0.929) in discriminating PET‐positive from PET‐negative patients. Using backward selection, we determined that a 2‐miRNA combination (miR‐127‐3p and miR‐24‐3p) yielded an optimal AUC of 0.872 with 79% specificity and 80% sensitivity (Figure 5a). Notably, the negative predictive value (NPV) of this combination outperforms the positive predictive value (PPV), 89% vs. 69% respectively, suggesting that low EV‐miRNA levels during treatment predicts absence of disease (Figure 5a).

When we combine the full EV‐miRNA panel with sTARC the AUC reaches 0.924 with a good sensitivity (96%) and high NPV of 97%. Based on backward selection, the optimal model for disease detection in this patient cohort was a combination of EV let‐7a‐5p and sTARC (AUC = 0.93, CI 0.87‐0.99, Figure 5a) with high sensitivity, specificity and 96% NPV. Bootstrap validation of the different models illustrates that there is minimal overfitting in the models, with the biggest over optimism estimate of 0.0039 (model: all miRNAs; Figure 5c). Consistent with their different biology, EV‐miRNAs and sTARC independently add to the prediction for the presence (PET‐positive) or absence (PET‐negative) of disease. Crucially, PD was not significantly different from pre‐treatment active samples (Supplemental Figure 8) again excluding treatment effects on the EV‐miRNA levels. Moreover, EV‐miR‐127‐3p or EV‐let‐7a‐5p in combination with sTARC discriminate PR from CMR. In conclusion, EV‐miRNAs and sTARC is a powerful treatment response biomarker combination with potential for identification of residual disease in patients with PR and identify patients with PD early during treatment.

## 4 DISCUSSION

A pressing clinical need for cHL patients is to personalize treatment by reducing the number of poly‐chemotherapy cycles (Borchmann et al., 2017; Jones et al., 2014) or incorporate immunotherapy to minimize toxicity while maintaining efficacy. FDG‐PET/CT is the principal method for response monitoring of cHL patients although liquid biopsies are considered as auxiliary tools, as interim FDG‐PET/CT has limitations and cannot be repeated frequently (Adams et al., 2015). This is the first study revealing that EV‐associated miRNAs have potential to detect residual lesions and combined with sTARC are useful as serial therapy response monitoring tool.

The clinical utility of circulating miRNAs for detection of cancerous lesions remains unclear in part because their cellular origin and dynamics has remained unclear. We found that EV‐based detection of circulating miRNAs is a robust method to detect residual lesions during treatment while low EV‐miRNA levels accurately determine ‘absence of disease’ which is potentially useful for decisions to reduce treatment (Borchmann et al., 2017). We found that EV‐miRNAs levels are stable for > 2‐3 years. Importantly, EV‐miRNA levels during treatment appear minorly affected by circulating blood cells (Figure 4d). Despite these encouraging observations, infection (or the related immune responses) may influence EV‐miRNA levels to some extent, as evidenced by the correlations between EV‐miRNAs, c‐reactive protein and erythrocyte sediment rate (Figure 4d) and observed in the patient example in Figure 3c. In addition, four miRNAs (i.e. let‐7a‐5p, miR‐24‐3p, miR‐21‐5p, miR‐127‐3p) are significantly increased in CMR patients with symptoms of the common cold (Supplemental Figure 7). The absolute difference in miRNA levels is however limited. Thus, mild symptoms of infection are unlikely a major contributor to EV‐miRNA levels in cHL patients. No correlation was found between EV‐miRNA levels and age, and in case of gender miR‐155‐5p and let‐7a‐5p is significantly elevated in males (Supplemental Figure 6). In contrast, sTARC, appears less suited due to stronger correlations with leucocyte and thrombocyte levels (Figure 4d) even though we confirmed that sTARC discriminates well between PET‐positive and PET‐negative disease. It cannot be excluded that during the treatment cycles effects on the bone‐marrow negatively influences sTARC measurements. Finally, sTARC‐levels differ between male and females (Supplemental Figure 6), as previously observed (Cuccaro et al., 2016).

Three of the five miRNAs in our panel support HRS cell survival *in vitro* (Figure 1e). Nevertheless, the rarity of HRS cells in the tumour mass and detectable levels of these miRNAs in plasma EVs in healthy donors and CMR suggests additional origins. Indeed, HRS‐specific EBV‐miRNAs could not be detected in EVs and only at low levels in protein fractions, which nevertheless disappeared after treatment (Figure 4e). Despite the uncertainty of cellular origin, the EV‐miRNA levels of our panel did not correlate with circulating blood cell counts, but rather with the presence of FDG‐PET detectable disease lesions during treatment, which is clinically relevant. Notably the EV‐miRNA levels remained surprisingly stable in post‐treatment follow up (Figure 4a) unless a relapse occurs (Figure 2b, 3c). In addition, the EV‐miRNA levels decreased early during treatment and remain low in virtually all CMR patients distinct from PR and PD (Supplemental Figure 8). It must be noted that our method of EV isolation with size‐exclusion chromatography has certain strengths and limitations as explained previously (van Eijndhoven et al., 2016). Despite possible impurities, the realization that blood‐samples from the same patient were drawn at any moment of the day without dietary constrictions over extended periods (up to 3 years), suggest (but does not rule out) that fluctuations in high‐density lipoprotein/low‐density lipoprotein do not majorly influence miRNA measurements in the EV‐enriched fractions. Promising complementary blood‐based biomarker strategies to sTARC and EV‐miRNAs (Spina et al., 2018) which appear to be secreted by viable tumour, is the detection of mutations or clone‐specific VDJ rearrangements in circulating tumour DNA (ctDNA), which is released when transformed (HRS) cells die. In other types of cancer these ctDNA‐based methods are sensitive and specific although clonal haematopoiesis may confound results to some extent (Kurtz et al., 2015; Spina et al., 2018). Other limitations are that these methods typically require larger blood volumes, are costly and a high turn‐around time make them less suitable for serial measurements (Kurtz et al., 2019).

Because the EV‐miRNAs and sTARC relate to different aspects of tumour biology i.e. support growth and attracting non‐malignant immune cells, combining these analytes may overcome limitations of a single analyte as recently demonstrated with ctDNA and protein biomarkers (Cohen et al., 2018). Notably let‐7a‐5p in combination with sTARC yielded a promising NPV of 96% with 94% sensitivity and 84% specificity to predict PET‐confirmed disease, including partial response and stable disease (Figure 5). However, this study is limited by the relative infrequency of non‐responding cHL patients, thus to what extent EV‐miRNAs, sTARC or ctDNA methods can predict outcome independently of PET still needs to be established in larger validation cohorts. To meet this challenge, an international research consortium has been formed that prospectively collects and aims to analyse longitudinal samples of high‐risk cHL patients for further validation on the clinical performance of blood‐based assays and compare their performance with iPET.

The results of this study show that a two‐analyte (EV‐miRNA/sTARC) assay is a cost‐effective method for serial prediction of FDG‐PET status in cHL. The EV‐miRNA/sTARC combination may be incorporated into future dynamic risk assessment models to guide personalized treatment for cHL patients that are currently at risk of severe long‐term side effects. Finally, our observations in a prospective cohort of longitudinal samples open new possibilities for circulating EV‐miRNAs as blood‐based biomarkers in cancer patients undergoing treatment.

## CONFLICT OF INTERESTS

Dirk Michiel Pegtel and Michael Hackenberg are co‐founders of Exbiome BV. Dirk Michiel Pegtel is CSO of ExBiome BV and has received travel compensation from Takeda.

## AUTHOR CONTRIBUTIONS

Conception and design: Esther E. E. Drees, Daphne de Jong, Josée M. Zijlstra, and D. Michiel Pegtel designed the study with the help of Monique A. J. van Eijndhoven and Nils J. Groenewegen. Provision of study and patient materials: Esther E. E. Drees, Leah I. Prins, Sandra A. W. M. Verkuijlen, Nils J. Groenewegen, Xuan‐Mai Tran, Jennifer Perez‐Boza, Monique A. J. van Eijndhoven and Josée M. Zijlstra. Collection and assembly of data: Esther E. E. Drees, Nils J. Groenewegen, Monique A. J. van Eijndhoven, Andrea Vallés‐Martí, Tessa Jasmijn Molenaar, Kevin Mol, Joey J. J. P. Karregat, Aikaterini Kalantidou, Margaretha G. M. Roemer, Phylicia Stathi, Gerben J. C. Zwezerijnen, Julia Driessen, Daphne de Jong, Josée M. Zijlstra and D. Michiel Pegtel. Data analysis and interpretation: Esther E. E. Drees, Margaretha G. M. Roemer, Catharina G. M. Groothuis‐Oudshoorn, Erik van Dijk, Bauke Ylstra, Ernesto Aparicio‐Puerta, Michael Hackenberg, Jennifer Perez‐Boza, Daphne de Jong, Josée M. Zijlstra and D. Michiel Pegtel. Manuscript writing: Esther E. E. Drees and D. Michiel Pegtel wrote the manuscript with contributions from Margaretha G. M. Roemer, Daphne de Jong and Josée M. Zijlstra.

Final approval of manuscript: All authors.

## Supporting information

Supporting information.

Supporting information.
