# Supplementary material for: Extracellular vesicle miRNA predict FDG‐PET status in patients with classical Hodgkin Lymphoma
Source: J Extracell Vesicles. 2021 Jul 15;10(9):e12121. doi: 10.1002/jev2.12121 (PMC8282992; doi:10.1002/jev2.12121)
Supplement: Supplementary file 1 — Supporting information. [file JEV2-10-e12121-s001.pptx]

## Slide 1
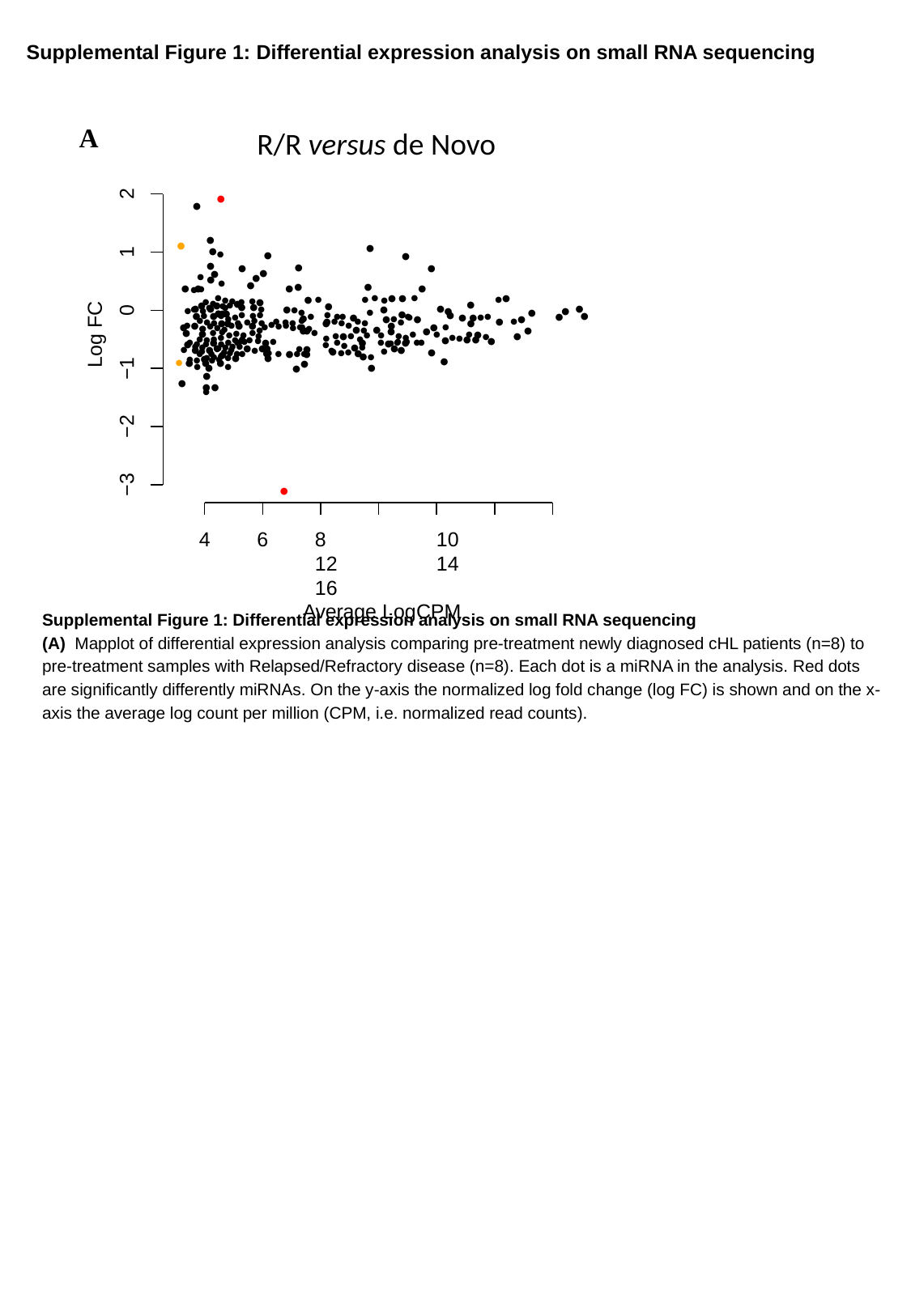

# Supplemental Figure 1: Differential expression analysis on small RNA sequencing
A
R/R versus de Novo
2
●
●
●
●
●●
●
1
●
●
●●
●
●
●
●
● ●
●
● ●
●
●● ●●●●
●
●
●
● ●
●
●
● ● ●● ● ●
● ●
●●● ● ●● ●●●
●● ●●●
● ●●	●
●●●●●●
●
● ●
●●●
●
Log FC
●
●
0
●
●
●● ●●●● ● ●●
● ●●●
● ●● ●
●
●●●
●●
●●●● ●
●
●
●●●● ●●
●
●●
●	●●●
●●●●● ● ●●● ● ●● ●
●●●●●●●●● ● ●●●●●●●● ●●●
●
● ●
●
●
●● ●
●●
● ●●●● ● ●
●
● ● ●●●● ●● ●●●●●
●●
●
● ●●● ●●
●
●●● ● ●
●
●●●● ●●● ●	●●●●●●
●● ●●●● ●● ●●● ●●●●●
● ●● ●
●● ● ● ●●
●
●
● ●● ●●
●●●●●●●●●
● ●
●●
● ●
● ●
● ● ●●●
●
● ●●●●
●
●
●
●	●●
●
●
●
●
● ●●● ●●
●
−1
●
●
●
●●
●
−2
−3
●
4
6
8	10	12	14	16
Average LogCPM
Supplemental Figure 1: Differential expression analysis on small RNA sequencing
(A) Mapplot of differential expression analysis comparing pre-treatment newly diagnosed cHL patients (n=8) to pre-treatment samples with Relapsed/Refractory disease (n=8). Each dot is a miRNA in the analysis. Red dots are significantly differently miRNAs. On the y-axis the normalized log fold change (log FC) is shown and on the x-axis the average log count per million (CPM, i.e. normalized read counts).

## Slide 2
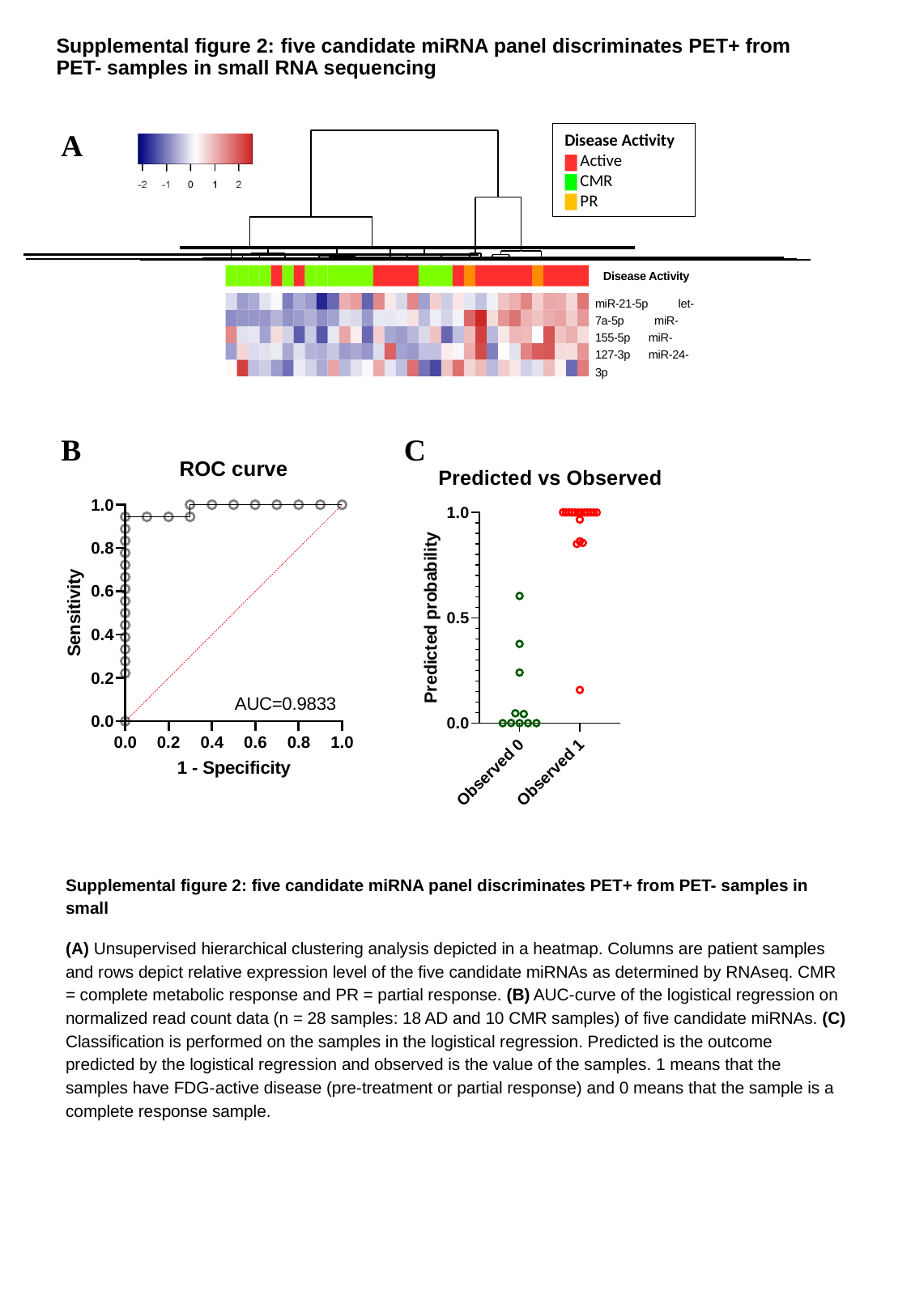

# Supplemental figure 2: five candidate miRNA panel discriminates PET+ from PET- samples in small RNA sequencing
A
Disease Activity
miR-21-5p let-7a-5p miR-155-5p miR-127-3p miR-24-3p
Disease Activity
 Active
 CMR
 PR
B		 C
Supplemental figure 2: five candidate miRNA panel discriminates PET+ from PET- samples in small
(A) Unsupervised hierarchical clustering analysis depicted in a heatmap. Columns are patient samples and rows depict relative expression level of the five candidate miRNAs as determined by RNAseq. CMR = complete metabolic response and PR = partial response. (B) AUC-curve of the logistical regression on normalized read count data (n = 28 samples: 18 AD and 10 CMR samples) of five candidate miRNAs. (C) Classification is performed on the samples in the logistical regression. Predicted is the outcome predicted by the logistical regression and observed is the value of the samples. 1 means that the samples have FDG-active disease (pre-treatment or partial response) and 0 means that the sample is a complete response sample.

## Slide 3
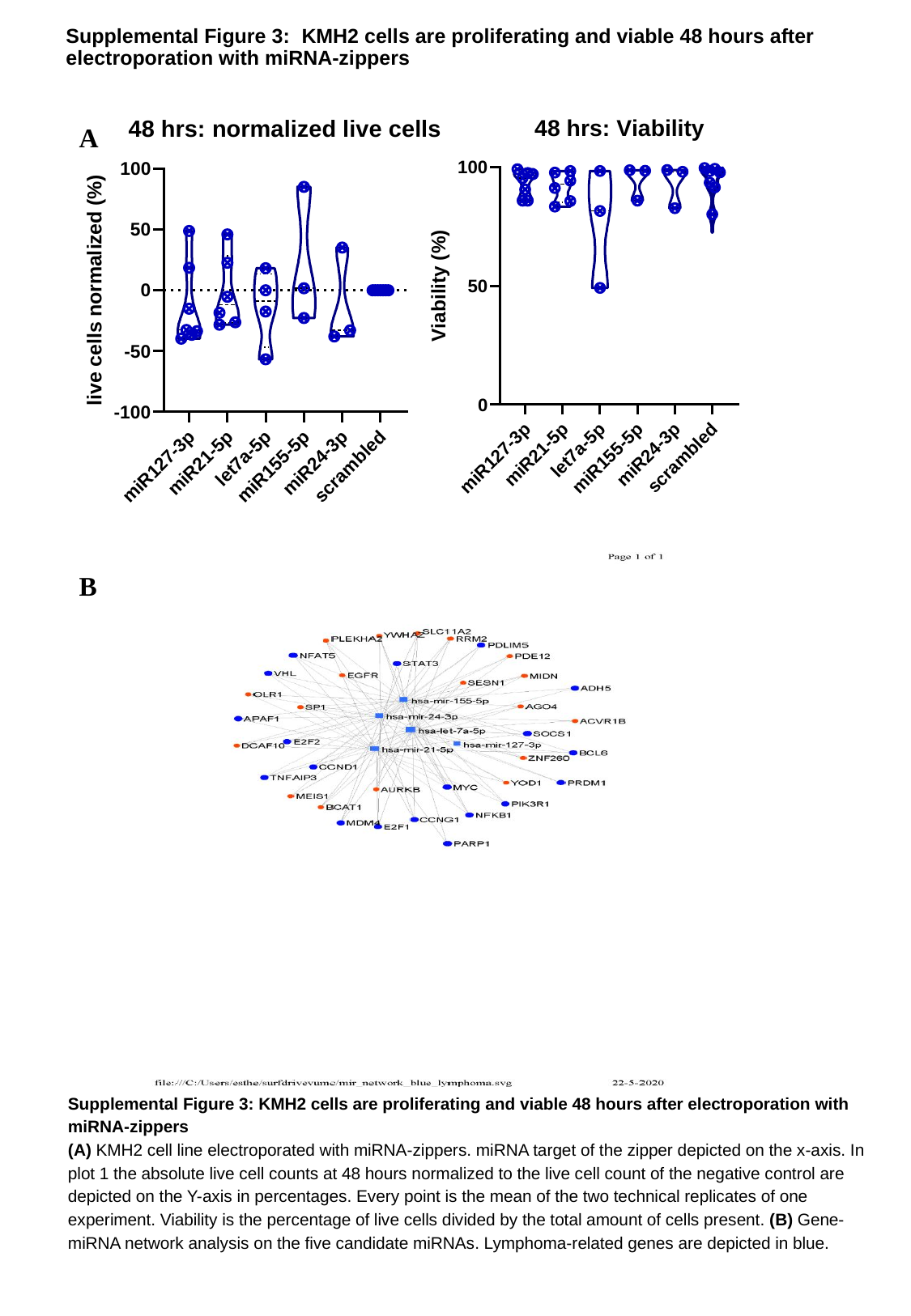

# Supplemental Figure 3: KMH2 cells are proliferating and viable 48 hours after electroporation with miRNA-zippers
A
B
Supplemental Figure 3: KMH2 cells are proliferating and viable 48 hours after electroporation with miRNA-zippers
(A) KMH2 cell line electroporated with miRNA-zippers. miRNA target of the zipper depicted on the x-axis. In plot 1 the absolute live cell counts at 48 hours normalized to the live cell count of the negative control are depicted on the Y-axis in percentages. Every point is the mean of the two technical replicates of one experiment. Viability is the percentage of live cells divided by the total amount of cells present. (B) Gene-miRNA network analysis on the five candidate miRNAs. Lymphoma-related genes are depicted in blue.

## Slide 4
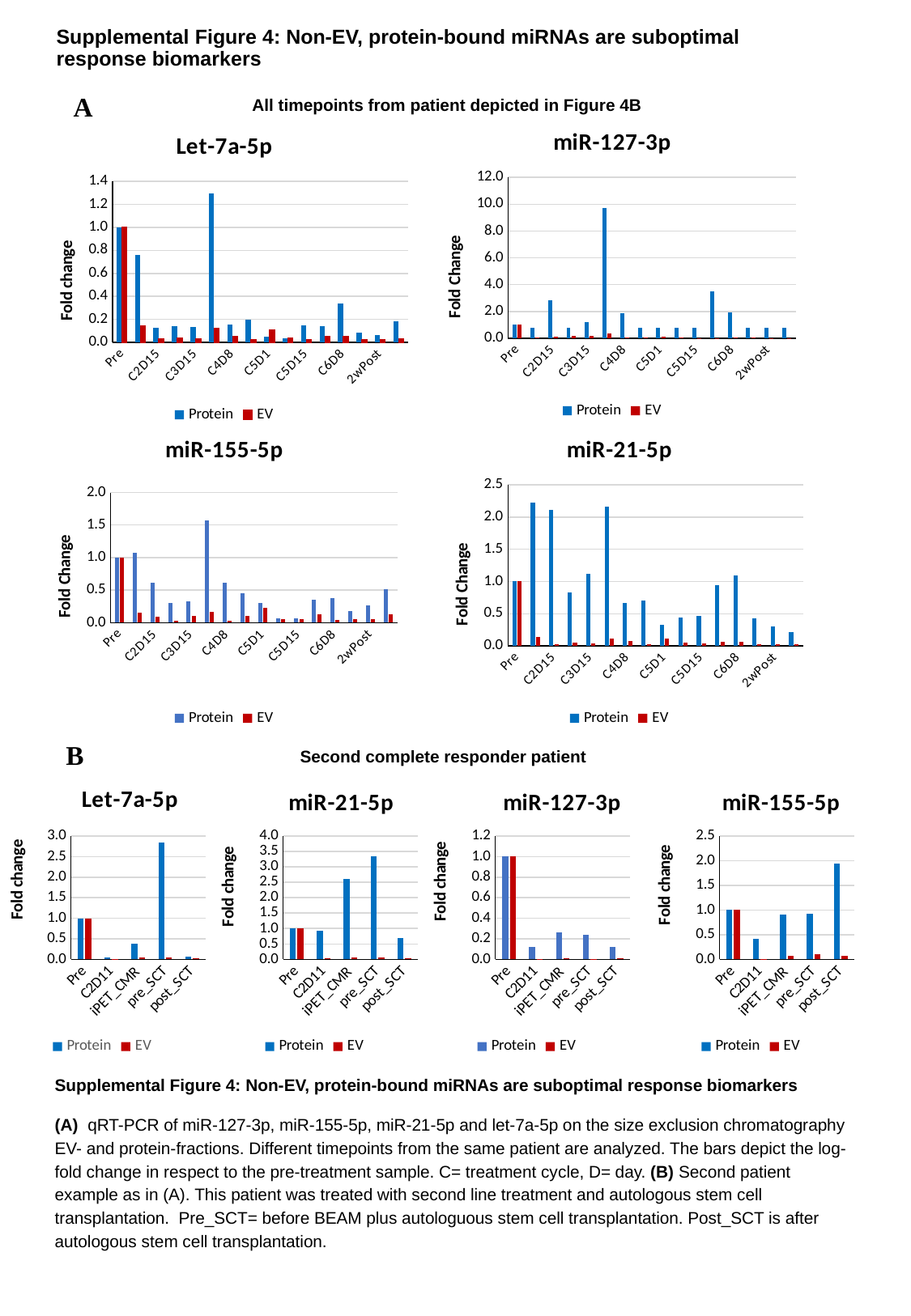

Supplemental Figure 4: Non-EV, protein-bound miRNAs are suboptimal response biomarkers
A
All timepoints from patient depicted in Figure 4B
### Chart: miR-127-3p
| Category | | |
|---|---|---|
| Pre | 1.0 | 1.0 |
| C2D1 | 0.7865026028042446 | 0.05457699163967095 |
| C2D15 | 2.849458592608805 | 0.11574386000510341 |
| C3D8 | 0.7865026028042446 | 0.16473501111369565 |
| C3D15 | 1.2311991040428436 | 0.1620030697529615 |
| C4D1 | 9.6876637823216 | 0.34558066842727225 |
| C4D8 | 1.868675011256711 | 0.06566164248678583 |
| C4D15 | 0.7865026028042446 | 0.05950557714356942 |
| C5D1 | 0.7865026028042446 | 0.12488321635738965 |
| C5D8 | 0.7865026028042446 | 0.05187519893385838 |
| C5D15 | 0.7865026028042446 | 0.022975287586137712 |
| C6D1 | 3.471999717613422 | 0.008821423315102567 |
| C6D8 | 1.9616515358262534 | 0.05775972087306555 |
| C6D15 | 0.7865026028042446 | 0.07709367486029058 |
| 2wPost | 0.7865026028042446 | 0.016102648116039366 |
| 5mPost | 0.7865026028042446 | 0.010929355297420658 |
### Chart: Let-7a-5p
| Category | | |
|---|---|---|
| Pre | 1.0 | 1.0 |
| C2D1 | 0.7571784686036602 | 0.1472878722689948 |
| C2D15 | 0.12974038169185112 | 0.032870734453542695 |
| C3D8 | 0.14389427818549405 | 0.03712238244421837 |
| C3D15 | 0.1342541684190318 | 0.03302820940279448 |
| C4D1 | 1.2931910608901538 | 0.12452232448302968 |
| C4D8 | 0.1519774325775909 | 0.05601489165206255 |
| C4D15 | 0.19372422307637355 | 0.027914862819666278 |
| C5D1 | 0.047996274528756155 | 0.10835019470931824 |
| C5D8 | 0.0350953389671553 | 0.03578880066712569 |
| C5D15 | 0.14670553196491962 | 0.02623007879238407 |
| C6D1 | 0.13933610127883014 | 0.05270155058428413 |
| C6D8 | 0.34061338784147904 | 0.05092895403445266 |
| C6D15 | 0.08733231173801759 | 0.024360731986612746 |
| 2wPost | 0.06511895091116165 | 0.02178805183865025 |
| 5mPost | 0.1854315046433137 | 0.029499237614064963 |
### Chart: miR-155-5p
| Category | | |
|---|---|---|
| Pre | 1.0 | 1.0 |
| C2D1 | 1.0782916797002364 | 0.15675153081474213 |
| C2D15 | 0.6085828005808676 | 0.09036100577171342 |
| C3D8 | 0.30169805313282794 | 0.034310403113643306 |
| C3D15 | 0.32888663960694225 | 0.11131440245213334 |
| C4D1 | 1.567334945615959 | 0.16779584980501924 |
| C4D8 | 0.614565994728657 | 0.03174263300470752 |
| C4D15 | 0.4576341975925021 | 0.10833200387990394 |
| C5D1 | 0.30009965490889834 | 0.22910330366547715 |
| C5D8 | 0.0684244284347245 | 0.058266612485035084 |
| C5D15 | 0.0684244284347245 | 0.0513362685567113 |
| C6D1 | 0.3484959305272428 | 0.1292796245274017 |
| C6D8 | 0.3741985312609121 | 0.04428605950463208 |
| C6D15 | 0.17854061284284603 | 0.05760773056543512 |
| 2wPost | 0.26184653914650513 | 0.059465940299419796 |
| 5mPost | 0.5133580452865437 | 0.13015424724908203 |
### Chart: miR-21-5p
| Category | | |
|---|---|---|
| Pre | 1.0 | 1.0 |
| C2D1 | 2.2257493784455566 | 0.13365884665429584 |
| C2D15 | 2.106508733247221 | 0.02001052843122806 |
| C3D8 | 0.8252937074862022 | 0.05120220999918599 |
| C3D15 | 1.1148221880907967 | 0.04349351054461908 |
| C4D1 | 2.1671642306104593 | 0.11660303753054113 |
| C4D8 | 0.6707686895523218 | 0.07457284129301348 |
| C4D15 | 0.7059858541724321 | 0.027649983541817063 |
| C5D1 | 0.32273683183303187 | 0.1101291561969087 |
| C5D8 | 0.43725540372237787 | 0.048854626976368914 |
| C5D15 | 0.4614654396664845 | 0.032905649373113584 |
| C6D1 | 0.9384078898927973 | 0.06213448683961565 |
| C6D8 | 1.091559261345954 | 0.06096478329028848 |
| C6D15 | 0.4296241517256244 | 0.028924700959062997 |
| 2wPost | 0.30009291017095624 | 0.021162886760656845 |
| 5mPost | 0.2175089471802718 | 0.027149340558960684 |B
Second complete responder patient
### Chart: Let-7a-5p
| Category | Protein | EV |
|---|---|---|
| Pre | 1.0 | 1.0 |
| C2D11 | 0.052370833607544715 | 0.014226486404563398 |
| iPET_CMR | 0.38002343905408026 | 0.05018749258446571 |
| pre_SCT | 2.849292840928945 | 0.052909872373743214 |
| post_SCT | 0.06862763609956164 | 0.02786368496283258 |
### Chart: miR-21-5p
| Category | Protein | EV |
|---|---|---|
| Pre | 1.0 | 1.0 |
| C2D11 | 0.9251738551490016 | 0.02572613040407062 |
| iPET_CMR | 2.609800889702901 | 0.0564113371422872 |
| pre_SCT | 3.338830117012799 | 0.07025847489989143 |
| post_SCT | 0.6844659189261895 | 0.03604361889982334 |
### Chart: miR-127-3p
| Category | Protein | EV |
|---|---|---|
| Pre | 1.0 | 1.0 |
| C2D11 | 0.11754751277360513 | 0.00511611731076415 |
| iPET_CMR | 0.2604475669900276 | 0.012048840658304754 |
| pre_SCT | 0.240404181887342 | 0.006283903958231671 |
| post_SCT | 0.11754751277360513 | 0.011133273155035407 |
### Chart: miR-155-5p
| Category | Protein | EV |
|---|---|---|
| Pre | 1.0 | 1.0 |
| C2D11 | 0.4215859288861565 | 0.011929416002446307 |
| iPET_CMR | 0.9101340790994069 | 0.07572756132952292 |
| pre_SCT | 0.9316251076678898 | 0.10592290237358049 |
| post_SCT | 1.9453279492414737 | 0.07779101067666284 |Supplemental Figure 4: Non-EV, protein-bound miRNAs are suboptimal response biomarkers
(A) qRT-PCR of miR-127-3p, miR-155-5p, miR-21-5p and let-7a-5p on the size exclusion chromatography EV- and protein-fractions. Different timepoints from the same patient are analyzed. The bars depict the log-fold change in respect to the pre-treatment sample. C= treatment cycle, D= day. (B) Second patient example as in (A). This patient was treated with second line treatment and autologous stem cell transplantation. Pre_SCT= before BEAM plus autologuous stem cell transplantation. Post_SCT is after autologous stem cell transplantation.

## Slide 5
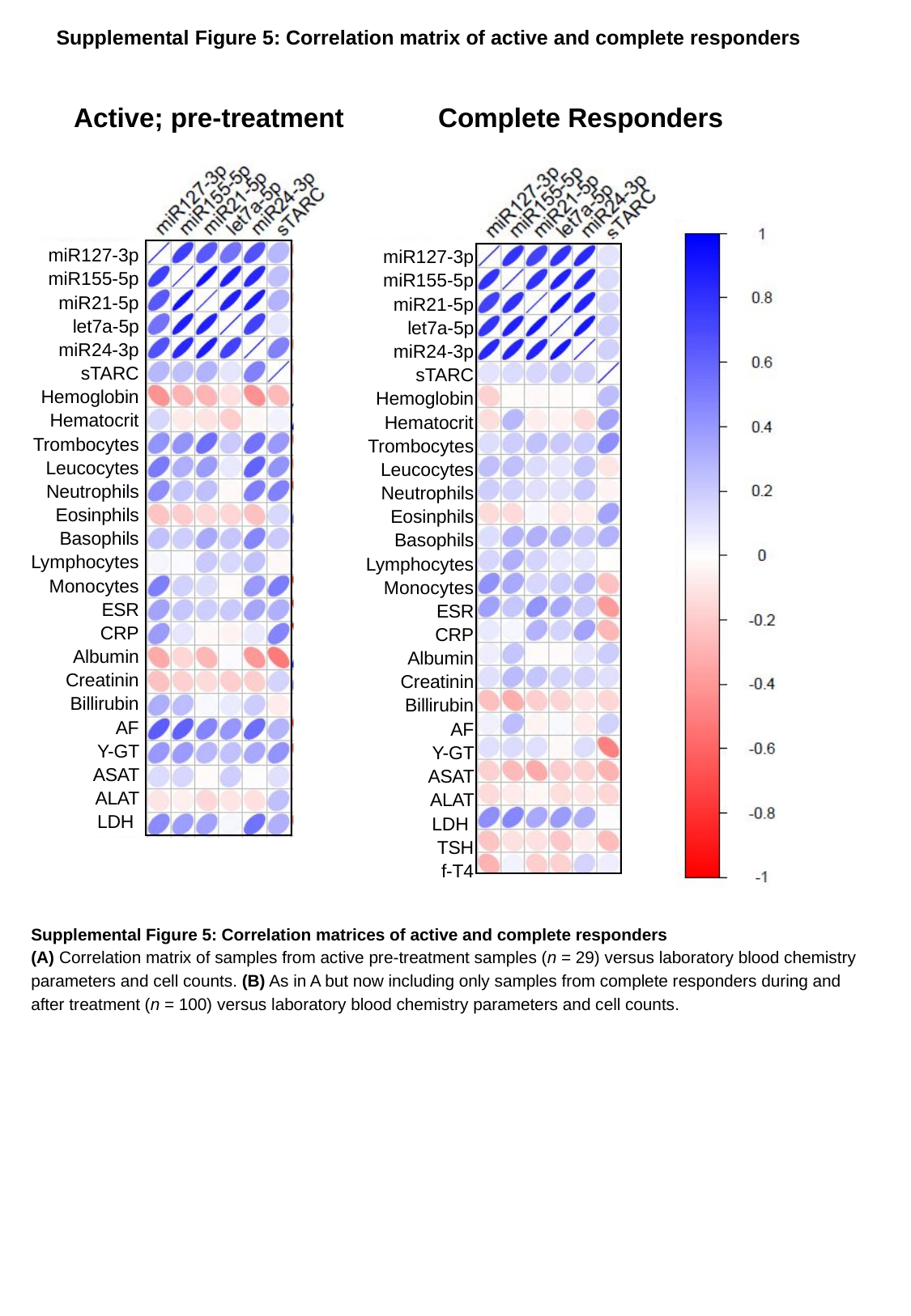

# Supplemental Figure 5: Correlation matrix of active and complete responders
Active; pre-treatment	Complete Responders
miR127-3p miR155-5p miR21-5p let7a-5p miR24-3p sTARC
Hemoglobin
Hematocrit
Trombocytes Leucocytes Neutrophils Eosinphils Basophils Lymphocytes Monocytes
ESR CRP
Albumin Creatinin Billirubin
AF
 Y-GT ASAT ALAT LDH
miR127-3p miR155-5p miR21-5p let7a-5p miR24-3p sTARC
Hemoglobin
Hematocrit
Trombocytes Leucocytes Neutrophils Eosinphils Basophils Lymphocytes Monocytes
ESR CRP
Albumin Creatinin Billirubin
AF
 Y-GT ASAT ALAT LDH
TSH
f-T4
ESR
ESR
Supplemental Figure 5: Correlation matrices of active and complete responders
(A) Correlation matrix of samples from active pre-treatment samples (n = 29) versus laboratory blood chemistry parameters and cell counts. (B) As in A but now including only samples from complete responders during and after treatment (n = 100) versus laboratory blood chemistry parameters and cell counts.

## Slide 6
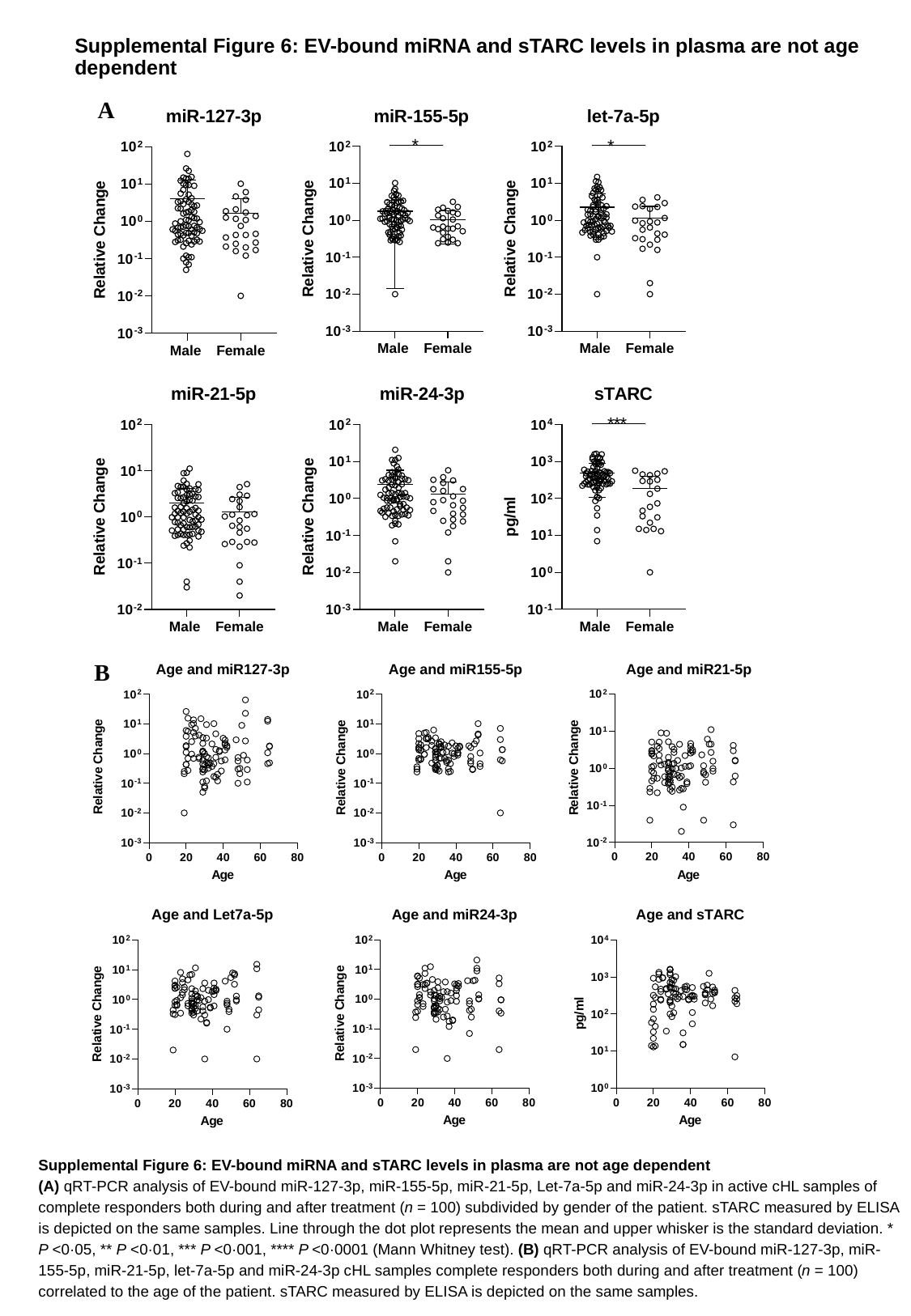

Supplemental Figure 6: EV-bound miRNA and sTARC levels in plasma are not age dependent
A
B
Supplemental Figure 6: EV-bound miRNA and sTARC levels in plasma are not age dependent
(A) qRT-PCR analysis of EV-bound miR-127-3p, miR-155-5p, miR-21-5p, Let-7a-5p and miR-24-3p in active cHL samples of complete responders both during and after treatment (n = 100) subdivided by gender of the patient. sTARC measured by ELISA is depicted on the same samples. Line through the dot plot represents the mean and upper whisker is the standard deviation. * P <0·05, ** P <0·01, *** P <0·001, **** P <0·0001 (Mann Whitney test). (B) qRT-PCR analysis of EV-bound miR-127-3p, miR-155-5p, miR-21-5p, let-7a-5p and miR-24-3p cHL samples complete responders both during and after treatment (n = 100) correlated to the age of the patient. sTARC measured by ELISA is depicted on the same samples.

## Slide 7
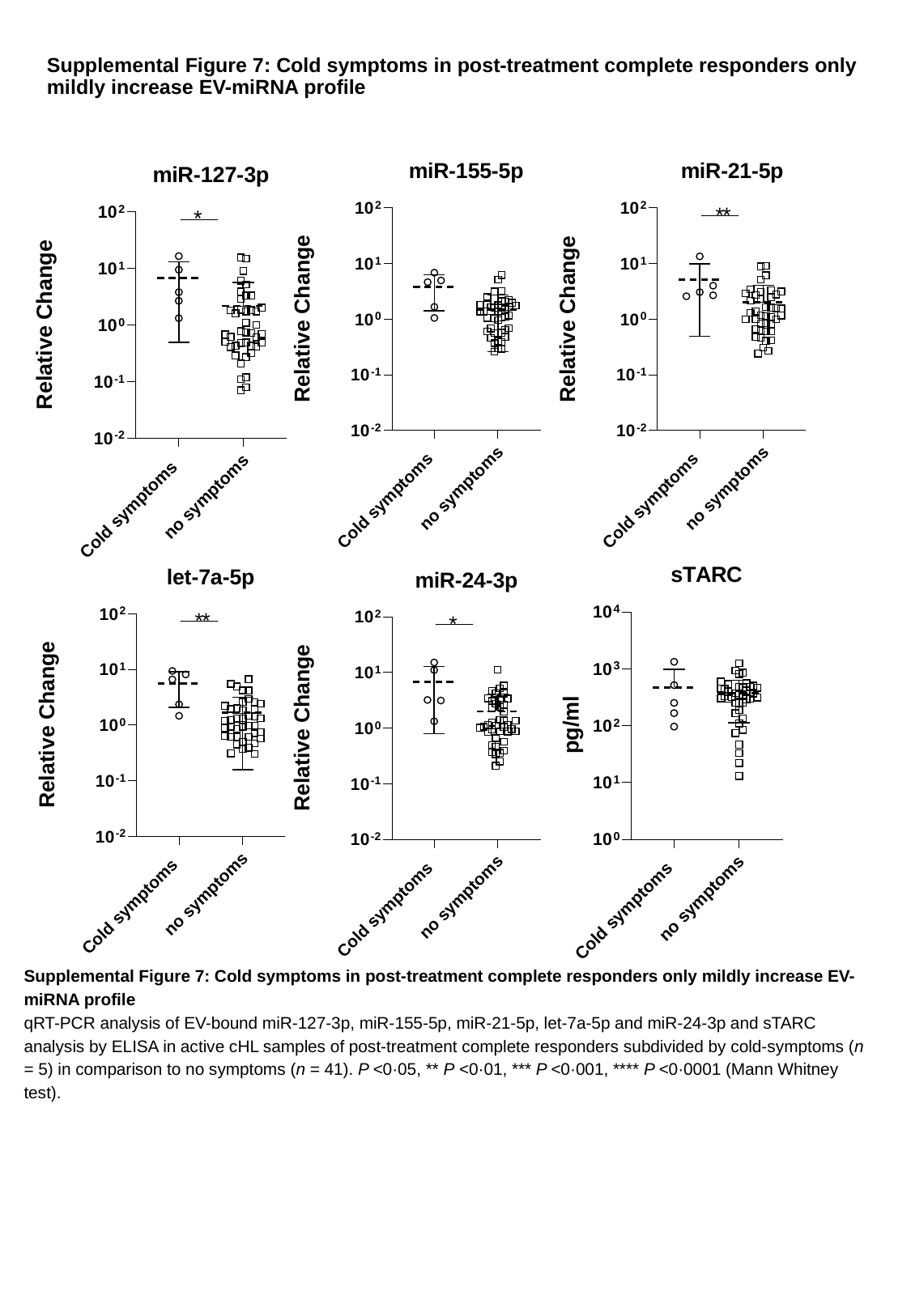

# Supplemental Figure 7: Cold symptoms in post-treatment complete responders only mildly increase EV-miRNA profile
Supplemental Figure 7: Cold symptoms in post-treatment complete responders only mildly increase EV-miRNA profile
qRT-PCR analysis of EV-bound miR-127-3p, miR-155-5p, miR-21-5p, let-7a-5p and miR-24-3p and sTARC analysis by ELISA in active cHL samples of post-treatment complete responders subdivided by cold-symptoms (n = 5) in comparison to no symptoms (n = 41). P <0·05, ** P <0·01, *** P <0·001, **** P <0·0001 (Mann Whitney test).

## Slide 8
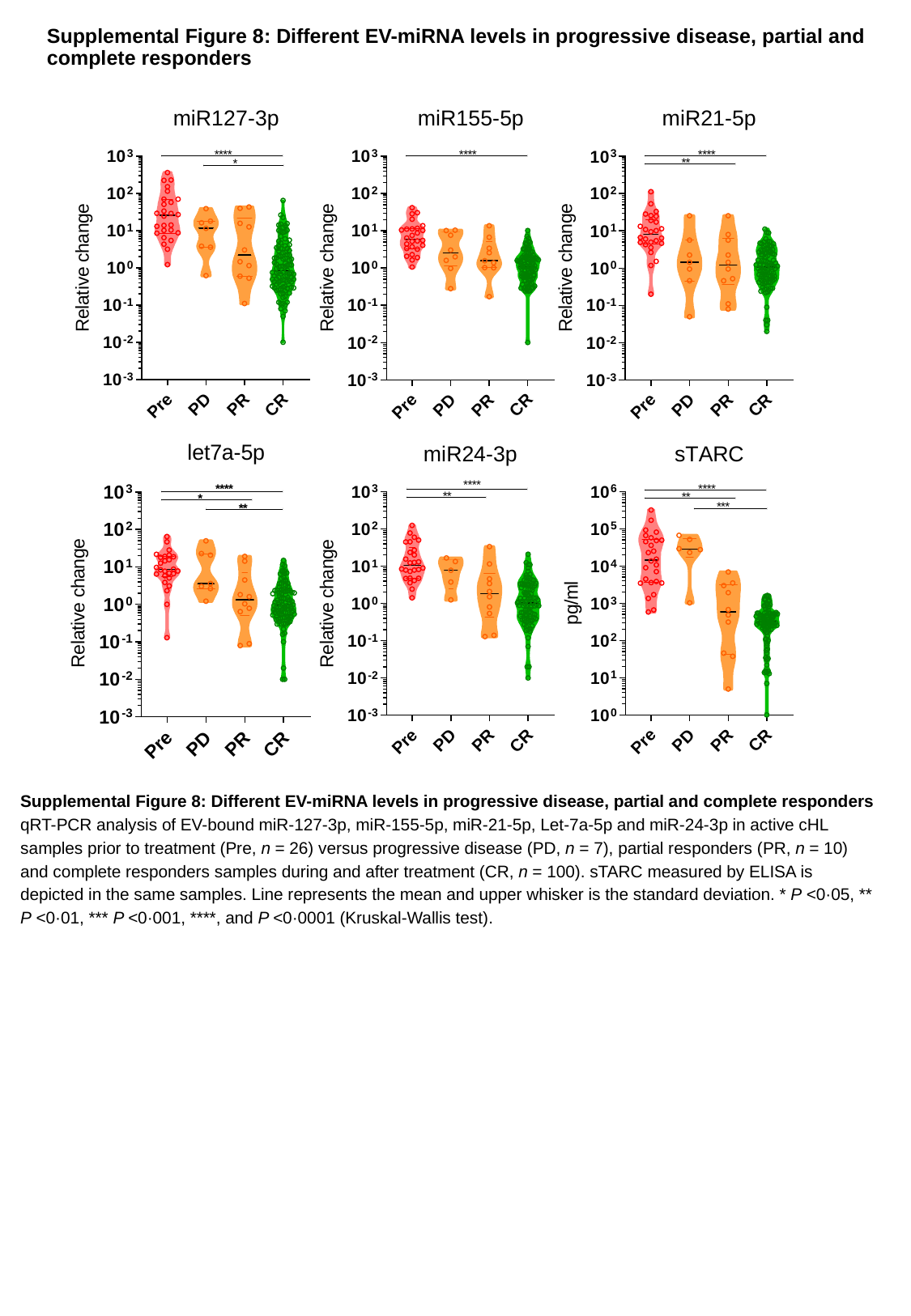

# Supplemental Figure 8: Different EV-miRNA levels in progressive disease, partial and complete responders
Supplemental Figure 8: Different EV-miRNA levels in progressive disease, partial and complete responders
qRT-PCR analysis of EV-bound miR-127-3p, miR-155-5p, miR-21-5p, Let-7a-5p and miR-24-3p in active cHL samples prior to treatment (Pre, n = 26) versus progressive disease (PD, n = 7), partial responders (PR, n = 10) and complete responders samples during and after treatment (CR, n = 100). sTARC measured by ELISA is depicted in the same samples. Line represents the mean and upper whisker is the standard deviation. * P <0·05, ** P <0·01, *** P <0·001, ****, and P <0·0001 (Kruskal-Wallis test).

## Slide 9
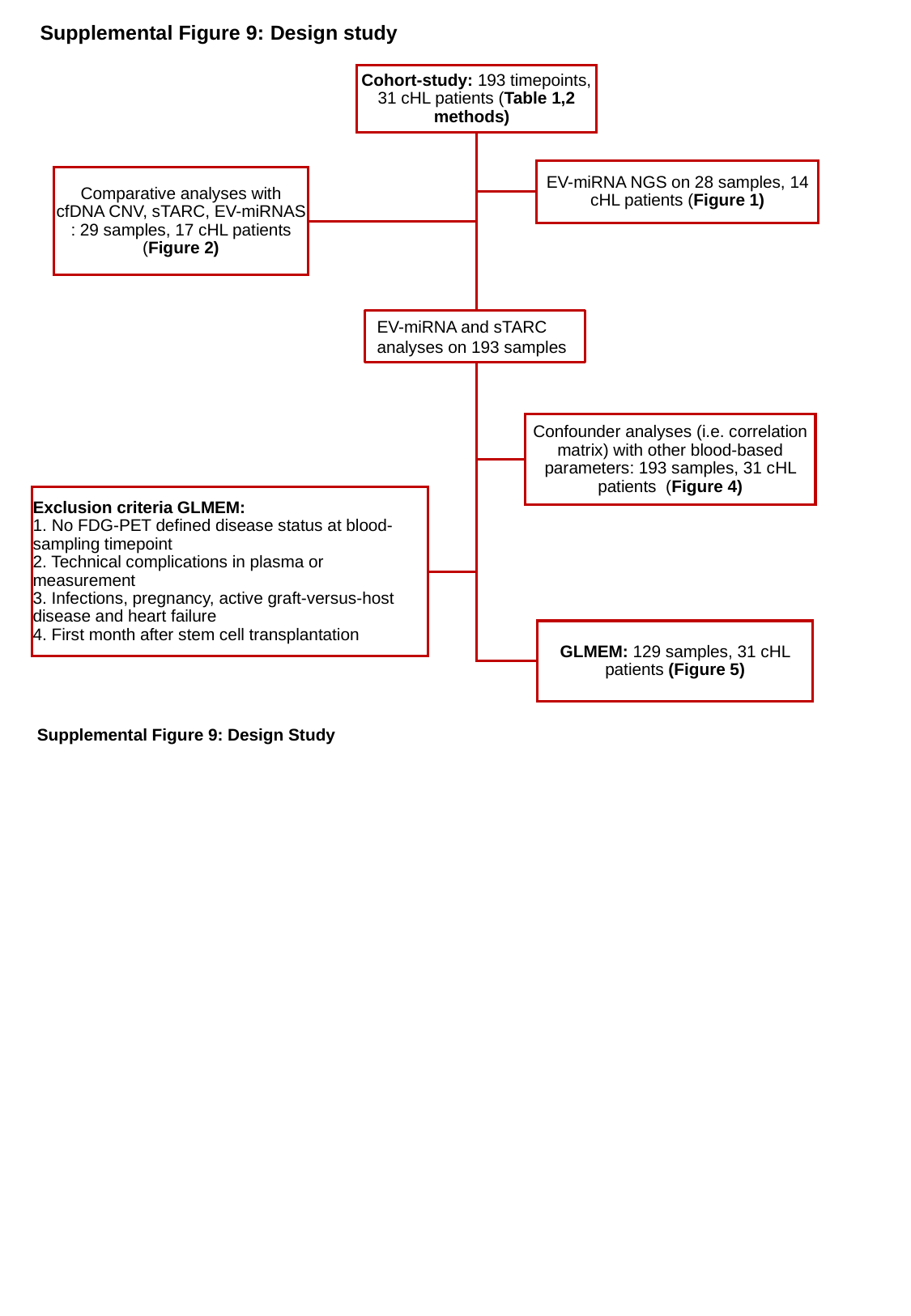

# Supplemental Figure 9: Design study
EV-miRNA and sTARC analyses on 193 samples
Supplemental Figure 9: Design Study

## Slide 10
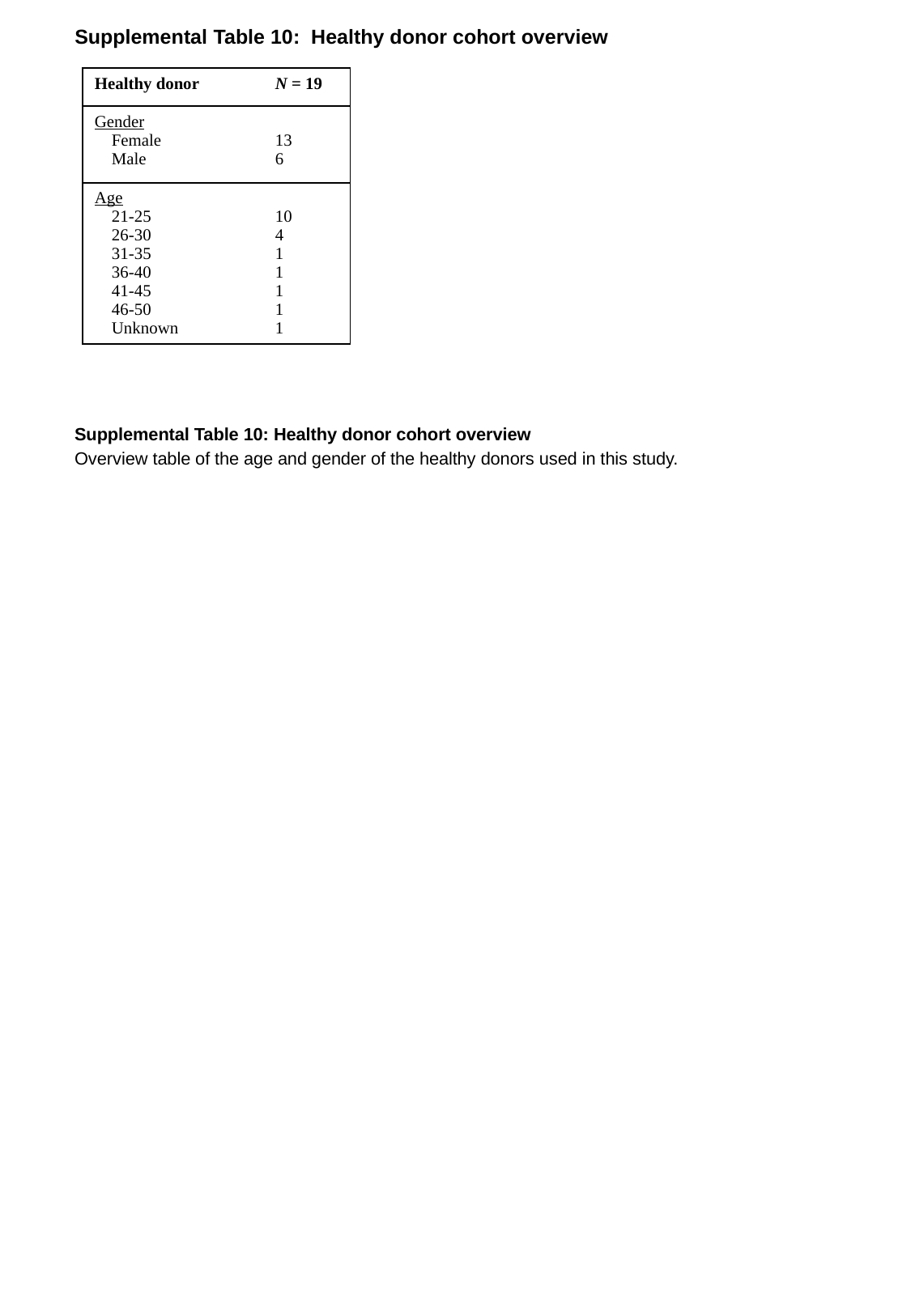

# Supplemental Table 10: Healthy donor cohort overview
| Healthy donor | N = 19 |
| --- | --- |
| Gender Female Male | 13 6 |
| Age 21-25 26-30 31-35 36-40 41-45 46-50 Unknown | 10 4 1 1 1 1 1 |
Supplemental Table 10: Healthy donor cohort overview
Overview table of the age and gender of the healthy donors used in this study.

## Slide 11
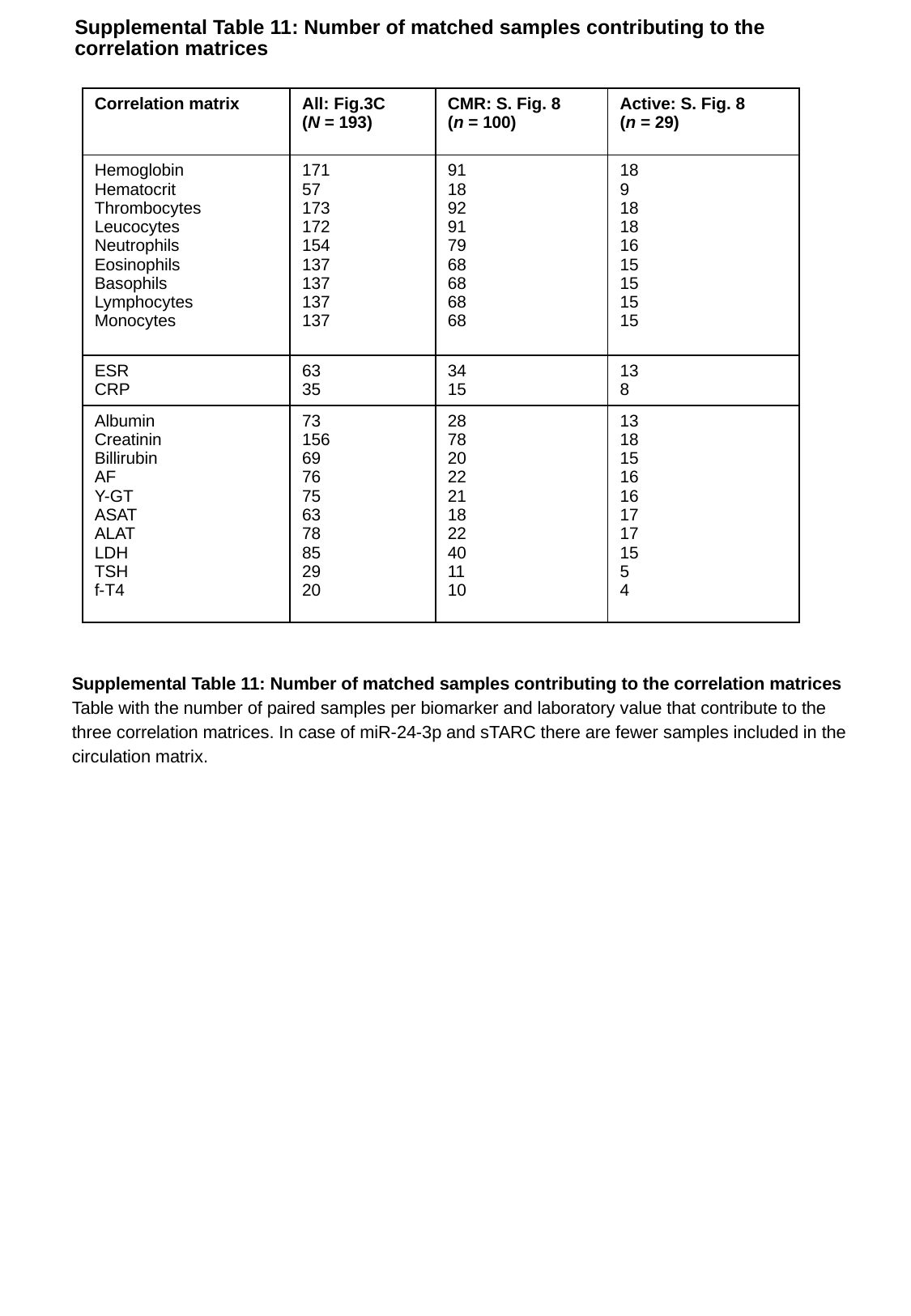

# Supplemental Table 11: Number of matched samples contributing to the correlation matrices
| Correlation matrix | All: Fig.3C (N = 193) | CMR: S. Fig. 8 (n = 100) | Active: S. Fig. 8 (n = 29) |
| --- | --- | --- | --- |
| Hemoglobin Hematocrit Thrombocytes Leucocytes Neutrophils Eosinophils Basophils Lymphocytes Monocytes | 171 57 173 172 154 137 137 137 137 | 91 18 92 91 79 68 68 68 68 | 18 9 18 18 16 15 15 15 15 |
| ESR CRP | 63 35 | 34 15 | 13 8 |
| Albumin Creatinin Billirubin AF Y-GT ASAT ALAT LDH TSH f-T4 | 73 156 69 76 75 63 78 85 29 20 | 28 78 20 22 21 18 22 40 11 10 | 13 18 15 16 16 17 17 15 5 4 |
Supplemental Table 11: Number of matched samples contributing to the correlation matrices
Table with the number of paired samples per biomarker and laboratory value that contribute to the three correlation matrices. In case of miR-24-3p and sTARC there are fewer samples included in the circulation matrix.
